# Pathogenic tau modifications occur in axons before the somatodendritic compartment in mossy fiber and Schaffer collateral pathways

**DOI:** 10.1186/s40478-019-0675-9

**Published:** 2019-02-28

**Authors:** Kyle R. Christensen, Thomas G. Beach, Geidy E. Serrano, Nicholas M. Kanaan

**Affiliations:** 1 Department of Translational Science and Molecular Medicine, Michigan State University, College of Human Medicine, 400 Monroe Ave NW, Grand Rapids, MI 49053 USA; 2 Neuroscience Program, Michigan State University, East Lansing, MI USA; 3 Banner Sun Health Research Institute, Sun City, AZ USA; 4 Hauenstein Neuroscience Center, Mercy Health Saint Mary’s, Grand Rapids, MI USA

**Keywords:** AT8 phosphoepitope, Conformation, Phosphorylation, Alzheimer’s disease, Tauopathies, Axonal degeneration, Amyloid-β

## Abstract

The deposition of tau pathology in Alzheimer’s disease (AD) may occur first in axons of neurons and then progress back into the cell bodies to form neurofibrillary tangles, however, studies have not directly analyzed this relationship in relatively discrete circuits within the human hippocampus. In the early phases of tau deposition, both AT8 phosphorylation and exposure of the amino terminus of tau occurs in tauopathies, and these modifications are linked to mechanisms of synaptic and axonal dysfunction. Here, we examined the localization of these tau pathologies in well-characterized post-mortem human tissue samples from the hippocampus of 44 cases ranging between non-demented and mild cognitively impaired to capture a time at which intrahippocampal pathways show a range in the extent of tau deposition. The tissue sections were analyzed for AT8 (AT8 antibody), amino terminus exposure (TNT2 antibody), and amyloid-β (MOAB2 antibody) pathology in hippocampal strata containing the axons and neuronal cell bodies of the CA3-Schaffer collateral and dentate granule-mossy fiber pathways. We show that tau pathology first appears in the axonal compartment of affected neurons in the absence of observable tau pathology in the corresponding cell bodies in several cases. Additionally, deposition of tau in these intrahippocampal pathways was independent of the presence of Aβ plaques. We confirmed that the majority of tau pathology positive neuropil threads were axonal in origin and not dendritic using an axonal marker (i.e. SMI312 antibody) and somatodendritic marker (i.e. MAP2 antibody). Taken together, these results support the hypothesis that AT8 phosphorylation and amino terminus exposure are early pathological events and that the deposition of tau pathology, at least in the studied pathways, occurs first in the axonal compartment prior to observable pathology in the somata. These findings highlight the importance on targeting tau deposition, ideally in the initial phases of its deposition in axons.

**Electronic supplementary material:**

The online version of this article (10.1186/s40478-019-0675-9) contains supplementary material, which is available to authorized users.

## Introduction

Tau is a microtubule-associated protein involved in regulating axon integrity and function [25, 76]. Notably, tau aggregation and deposition are hallmarks of Alzheimer’s disease (AD) as well as numerous other tauopathies [14, 31, 36, 44, 53]. Pathological modifications and aggregation of tau associate with cognitive decline [23, 29, 30]. The deposition of amyloid-β (Aβ) in plaques represents the other hallmark AD pathology and likely contributes to neurotoxicity in AD [45, 53]. Alterations in synapse morphology, synapse loss, and axon degeneration occur early in the progression of AD [8, 23, 52, 75]. This has led to the hypothesis of a “dying-back” pattern of degeneration, where axon degeneration precedes loss of cell bodies [42]. The hypothesis that tau pathology begins in the axonal compartment and appears in the somatodendritic compartment afterwards is often suggested, however, a direct analysis of axon enriched layers in the hippocampal formation has not previously been conducted [29, 46]. Moreover, the amyloid cascade hypothesis suggests that Aβ pathology precedes and induces the accumulation of tau pathology presumably starting in axonal target regions of neurons affected by tau [34, 35, 69]. Animal models of tauopathy also support the contention that tau deposition occurs in the synaptic and axonal compartments prior to the somatodendritic compartment [4, 22]. Observations in human tissue describing the appearance of extensive neuropil threads (NTs) before detection of neurofibrillary tangles (NFTs) in neuronal cell bodies within a given neuroanatomical region support this hypothesis, though neuropil threads can represent both axons and dendrites [29, 66–68, 71, 75]. None of these studies assessed the terminal fields and somata of specific pathways in the earliest stages of tau deposition precluding a clear determination of whether axonal pathology precedes cell body pathology in cells.

During the course of tau deposition in disease the tau proteins undergo fairly well-characterized changes including both the morphology of and the post-translational modifications to the tau proteins within the pathology [5, 11, 18, 33, 56]. Importantly, there are specific changes that occur during the earliest detectable deposition of tau inclusions in human brains. These are often referred to as pretangle markers for their ability to recognize tau pathology prior to its maturation and coalescence into compact tau inclusions [5, 12, 13, 15, 18]. For example, AT8, a triple phosphoepitope including phospho-S199/S202/T205, appears early in diffuse granular pretangle inclusions in neurons [9, 32]. Additionally, conformational display of an N-terminal region known as the phosphatase-activating domain (PAD), a change recognized by the TNT2 antibody, occurs in pretangle neurons in AD and several tauopathies [18, 19]. Importantly, AT8 causes an extension of the N-terminus of tau away from the microtubule binding repeats [38], and both AT8 and exposure of PAD are linked to a specific mechanism of tau toxicity involving impaired axonal function (i.e. axonal transport inhibition) [40, 41, 48]. Though these markers are modifications of tau that appear in the earliest detectable tau inclusions, it remained unclear whether these pathogenic forms of tau first appeared in axons before progressing to the neuronal cell bodies in humans.

The hippocampal formation comprises the entorhinal cortex (EC), dentate gyrus (DG), hippocampus proper (subdivided into CA1, CA2, CA3, and hilus), subiculum, presubiculum, and parasubiculum [2, 3]. The EC receives neocortical input and projects through the angular bundle and perforant path to terminate in the molecular layer of the DG [1, 3]. The DG granule cell projections, known as mossy fibers, terminate in the CA3 stratum lucidum layer (Str. Luc.). Next, CA3 pyramidal cell projections, known as Schaffer collaterals, terminate in the CA1 stratum radiatum layer (Str. Rad.). A majority of the CA1 pyramidal cell projections terminate in the subiculum, however, some also project back to the EC. Finally, most subiculum projections pass back through the angular bundle to the EC to complete the circuit [2, 3]. The well-defined intrahippocampal circuitry and relatively distinct strata provide an ideal structure to analyze the compartmental progressive deposition of pathological tau within discrete neuronal pathways in post-mortem human tissue.

Tau pathology in the form of NFTs and NTs follows a regional progression in severity, that was described by Braak and Braak in the early 1990’s (i.e. Braak staging) [12, 14]. Braak staging was originally developed based on silver staining of tau pathology and later adapted to AT8+ pathology, and the primary focus was on the emergence and distribution of NFTs and NTs, and specifically excluded the tau pathology within neuritic plaques (NPs) [12, 14]. Deposition of tau inclusions begins in the transentorhinal and EC at Braak stages I-II, which is not associated with cognitive decline [30, 50] or NFT pathology in the hippocampal pyramidal cells [12, 14]. The limbic stages (Braak III-IV) display spread of tau pathology into the hippocampal formation, initially including primarily the CA1 region, but it is not until stages V-VI that the entire hippocampal formation is affected [12, 14]. Cognitive decline occurs at these stages and patients may display criteria for mild cognitive impairment (MCI), a prodromal stage of AD [5, 28, 61, 62, 74]. Finally, the isocortical stage (Braak V-VI) displays extensive tau pathology throughout the hippocampal formation and subdivisions of the cerebral cortex. In the presence of threshold densities and distributions of neuritic and amyloid plaques, Braak stages III and IV are termed “intermediate” Alzheimer’s disease neuropathologic change (ADNC) while Braak stages V and VI are designated as “high” ADNC [57]. Although a “high” level of ADNC does not predict, with 100% certainty, the presence of dementia, it has been accepted by an international panel of neuropathologists [57] that “intermediate” and “high” ADNC are sufficient to cause cognitive impairment and data suggests the probability of Braak V or VI subjects having dementia is > 95% [64]. In addition, recent neuropathological examination suggested that brains containing AD-like NFT pathology, but without detectable Aβ pathology represent a condition termed primary age-related tauopathy (PART) [21]. PART is typically associated with cognitive status ranging from no impairment to MCI.

The current study addresses the hypothesis that tau pathology first deposits in the axonal compartment of neurons prior to its appearance in the somata. Using post-mortem human hippocampal sections from non-demented (ND) controls and MCI cases, the extent of local axonal and somatodendritic tau pathology (AT8 phosphorylated and PAD exposed), as well as local Aβ pathology in the CA3-Schaffer collateral and DG-mossy fiber pathways was measured. We found AT8 phosphorylation and PAD exposure occurs in the axon compartment of affected neurons even in the absence of observable cell body pathology. Additionally, these tau pathological modifications were observed in the absence of amyloid plaques. Interestingly, separation of cases into PART and non-PART groups revealed that PART cases have significantly less AT8 and TNT2 pathology in these two intrahippocampal pathways. Overall, our results support the hypothesis that tau pathology may begin in the axonal compartment and is observed independently of Aβ plaque deposition.

## Materials and methods

### Human brain tissues

Formalin-fixed temporal lobe free-floating sections (40 μm) from ND (*n* = 31) and MCI (*n* = 13) cases were obtained de-identified from the Banner Sun Health Research Institute Brain and Body Donation Program (Sun City, AZ) [7]. Most subjects received annual standardized cognitive assessments prior to death. If death occurred prior to a standard assessment, cognitive status was determined by postmortem informant telephone questionnaire and private medical records review followed by a consensus diagnostic conference of Program neuropsychologists and neurologists. Global neuropathological examination methodology was previously described [7] and included assignment of Braak stage, CERAD neuritic plaque density [55], and National Institute on Aging-Reagan Institute AD probability level (0 = not AD; 1 = low; 2 = intermediate) [57]. Semi-quantitative scores (0–3) for global amyloid plaque and neurofibrillary tangle densities were obtained for frontal, parietal, and temporal neocortex as well as entorhinal and hippocampus regions; the sum of all regional scores are termed “global plaque density” and “global tangle density” scores. Table 1 summarizes the clinical, demographic, and global measures of neuropathology. The donation program also features a standing 24–7 response team that allows exceptionally low post-mortem intervals (PMIs) with a median PMI of 3.2 h for all 1900+ cases collected since 1988 [7].

**Table 1:** Demographic, clinical, and neuropathological characteristics by diagnosis

	Clinical diagnosis			Comparison by diagnosis group
	ND	MCI	Total	(*P* value)
	(N = 31)	(N = 13)	(*N* = 44)	
Age at death (years)				
Mean ± SD	83.1 ± 6.1	86.2 ± 5.4	84.0 ± 6.0	0.13^#^
(Range)	(69–97)	(74–95)	(69–97)	
Sex	19 M/12F (61.3% Male)	8 M/5F (61.5% Male)	27 M/17F (61.4% Male)	> 0.99^‡^
Postmortem Interval (hours)				
Mean ± SD	2.7 ± 0.6	2.7 ± 0.5	2.7 ± 0.6	0.84^#^
(Range)	(1.5–4.8)	(1.8–3.5)	(1.5–4.8)	
MMSE				
Mean ± SD	28.5 ± 1.3	27.4 ± 2.4	28.2 ± 1.7	0.34^#^
(Range)	(26–30)	(23–30)	(23–30)	
No Score (N)	8	4	12	
Braak Stage				
I	5	3	8	0.85^§^
II	7	3	10	
III	19	7	26	
NIA-Reagan AD Probability Level				
Not AD (0)	9	4	13	0.87^§^
Low (1)	7	2	9	
Intermediate (2)	15	7	22	
High (3)	0	0	0	
Global CERAD Plaque Density				
None (0)	9	4	13^	0.96^§^
Sparse (1)	7	3	10^$^	
Moderate (2)	8	4	12^$^	
Frequent (3)	7	2	9^$^	

*ND* non-demented, *MCI* mild cognitive impairment, *MMSE* Mini-Mental State Examination, *NIA-Reagan* National Institute on Aging-Reagan Institute AD probability level, *CERAD* Consortium to Establish a Registry for Alzheimer’s disease, *AD* Alzheimer’s disease. ^primary age-related tauopathy (PART) cases; ^$^non-PART cases; ^#^Mann-Whitney test; ^‡^Fisher’s exact test; ^§^Chi-square test

### Tissue immunohistochemistry (IHC)

Temporal lobe sections were immunohistochemically stained as previously described [18, 40, 41] to visualize the pattern of AT8 phosphorylation, PAD exposure, and Aβ pathologies using the monoclonal AT8 (Thermo MN1020), TNT2 (Kanaan lab) [18, 19], and MOAB2 (Kanaan lab, originally created by Dr. Lester Binder at Northwestern) [77] antibodies, respectively. Primary antibodies were diluted in tris-buffered saline (TBS; 150 mM NaCl, 50 mM Tris, pH 7.4) containing 2% goat serum and 0.1% Triton X-100 at 1:16,000 for AT8, 1:400,000 for TNT2, and 1:4000 for MOAB2. Immunoreactivity was detected using biotinylated goat-anti-mouse IgG (H + L) secondary antibody (Jackson ImmunoResearch Laboratories 115–065-166) diluted in TBS + 2% goat serum + 0.1% Triton X-100, VectaStain Elite ABC-HRP Kit (Vector Laboratories PK-6100), and 3,3′-diaminobenzidine supplemented with 0.25% ammonium nickel (II) sulfate hexahydrate (Sigma A1827). All sections were counterstained with cresyl violet before being mounted on microscope slides and coverslipped with Cytoseal 60 (Thermo Scientific, #8310–16). Tissue sections from each case were processed simultaneously for each antibody to eliminate inter-run staining variability. Primary antibody delete controls were run using the same protocol with the exception that the primary antibody was omitted. As expected, the primary deletes produced no staining (Additional file 1: Figure S1).

### Stereological axon measurements and total neuron enumeration

The unbiased stereological spaceballs probe was used to estimate the total length of neurites in single hippocampal body sections from each case stained with AT8 and TNT2 in the CA3 Str. Luc. layer (i.e. mossy fibers) and the CA1 Str. Rad. layer (i.e. Schaffer collaterals). The CA3 Str. Luc. was defined using fiduciary neuroanatomical landmarks, including the CA3 pyramidal cell layer dorsally, Str. Rad. of CA3 ventrally, the CA2 medially, and hilus laterally. The CA3 pyramidal layer was defined using fiduciary neuroanatomical landmarks, including the CA3 Str. Luc. dorsally, stratum oriens ventrally, CA2 medially, and hilus laterally. The CA1 Str. Rad. was defined using fiduciary neuroanatomical landmarks, including the CA1 pyramidal cell layer dorsally, stratum lacunosum-moleculare ventrally, subiculum medially, and CA2 laterally. The DG granule cell layer was defined using fiduciary neuroanatomical landmarks, including the hilus dorsally and the molecular layer ventrally, and is clearly defined by cresyl violet staining due to cell density and size. If specific subregions were not reliably identifiable within the sections, the case was not used for analyses requiring that region (5, 2, 0, and 5 cases were excluded from the CA3 Str. Luc., DG, CA1 Str. Rad. and CA3 analyses, respectively). A hemisphere probe with a radius of 8 μm was used to sample sites throughout each region. Mounted tissue thicknesses ranged from ~ 11–14 μm (≥70% shrinkage in the z-plane is typical after similar processing of free-floating sections [24, 26, 54]) across all cases and regions analyzed. A 4x objective was used to outline each contour and a 60x oil immersion objective (numerical aperture = 1.35) was used for making the stereological measurements. Local neurite density was calculated by dividing the estimated total axon length by the volume of the region of analysis, and neurite density was used for comparisons. Local somata staining was quantified by total enumeration in the CA3 pyramidal cell layer (i.e. Schaffer collateral pathway) and DG layer (i.e. mossy fiber pathway) of the same sections from above for neurite analyses using 10x magnification and manually counting the cell bodies displaying immunoreactivity. Total cell numbers were used for comparisons. Brightfield images were acquired on a Nikon Eclipse 90i microscope equipped with a Nikon DS-Ri1 camera and processed using Nikon NIS-Elements software.

### Multi-label immunofluorescence (IF)

Hippocampal sections from a subset of the 44 cases (*n* = 12 cases with high and low tau pathology) were double labeled with TNT2 (mouse IgG1, 1:8000) and biotinylated AT8 (mouse IgG1-biotin, 1:800, Thermo Scientific MN1020B) using methods similar to those previously published [39, 73]. Each primary antibody incubation period was overnight at 4 °C. TNT2 immunoreactivity was detected using AlexaFluor 647-conjugated goat-anti-mouse IgG (H + L) Fab fragments (Jackson ImmunoResearch Laboratories 115–547-003 and 115–607-003) and AT8 immunoreactivity was detected using AlexaFluor 568-conjugated streptavidin (ThermoScientific Pierce S11226). Sections were blocked with unconjugated goat-anti-mouse IgG (H + L) Fab fragments (Jackson ImmunoResearch Laboratories 115–007-003) to prevent cross-labeling of the secondary antibodies between each primary and subsequent secondary antibody incubations. Nuclei were counterstained in the sections by including DAPI (1 μg/ml; ThermoFisher, D1306) in the first rinse after the last detection secondary antibody step. Control sections included omission of each individual primary antibody. As expected, the individual primary delete sections did not produce cross-reaction of signals in the deleted antibody channel (Additional file 2: Figure S2).

In a second and third staining series, hippocampal sections were triple-labeled with either biotinylated AT8 (as above) or TNT2 (as above) and both SMI-312 (mouse IgG1, 1:1000, Biolegend 837,904) and MAP2 (rabbit polyclonal, 1:300, Cell Signaling 8707) to colocalize each of these tau pathologies with an axonal (SMI-312) and dendritic (MAP2) marker. All tissues were stained using a similar protocol to those previously published [39, 73]. Briefly, all sections were incubated overnight at 4 °C in SMI-312 and MAP2 primary antibodies and the following day these primaries were labeled with AlexaFlour 568 goat anti-mouse IgG (H + L) (1:500; ThermoFisher, A11031) and AlexaFlour 647 goat anti-rabbit (1:500; ThermoFisher, A21245) secondary antibodies, respectively. The tissue sections used for the TNT2/SMI-312/MAP2 series were blocked in 2% mouse serum (Invitrogen, 10,410) to saturate open binding sites on the first anti-mouse secondary antibody) for 1 h, followed by an hour incubation in goat anti-mouse whole molecule (1:50, Jackson ImmunoResearch, 115–008-003) to block binding sites on mouse IgGs. After blocking was completed, the tissue sections were incubated in TNT2 primary antibody overnight at 4 °C. The AT8/SMI-312/MAP2 series sections were incubated in biotinylated AT8 antibody (biotinylation precludes the need for the above blocking) overnight at 4 °C. The following day the TNT2 or AT8-biotin primary antibodies were labeled with goat anti-mouse IgG1-specific AlexaFlour 488 (1:500; ThermoFisher, A21121) or streptavidin conjugated to AlexaFluor 488 (1:500; ThermoFisher, S11223). After immunolabeling the tissues were counterstained with DAPI as above before mounting and coverslipping. Control sections included omission of each tau primary antibody. As expected, omission of the primary antibodies did not produce cross-reaction of signals in the deleted antibody channel confirming the tau localization with SMI312 and MAP2 was due to specificity of tau labeling (Additional file 3: Figure S3). Colocalization between tau markers (AT8 or TNT2) and either SMI-312 or MAP2 in neurites within the CA3 Str. Luc. and CA1 Str. Rad. was determined using these sections.

After staining, sections were mounted on microscope slides and autofluoresence of the tissue was blocked by treating with 2% Sudan Black B before coverslipping with VectaShield Hard Set mounting medium (Vector Laboratories H-1000). All IF images were obtained using a Nikon A1+ scanning confocal microscope system. Z-stacks were acquired in 0.5 μm steps at 60x magnification and images for figures were generated with a maximum intensity projection or slices view (for cross-sectional analysis for colocalization) using NIS-Elements software.

### Statistical analyses

All data were analyzed using Prism v7.0 software (GraphPad). Stereological estimate outcomes were analyzed for normality using the D’Agostino and Pearson normality test. All data sets were not normally distributed, and subsequently, non-parametric statistical analyses were performed. Cell number and Aβ plaque data sets were Log(x + 1) transformed for correlations because values of zero were present. All correlation comparisons were performed using the Spearman rank correlation and significant *p*-values were adjusted to ≤0.005 from ≤0.05 because 10 comparisons were performed within a given pathway (i.e. DG-mossy or CA3-Schaffer) for each immunostain (i.e. AT8 or TNT2) (Tables 4 and 7). Demographic variables were compared between clinical diagnostic groups using Mann-Whitney, Fisher’s exact, or the Chi-squared test. Statistical significance was set at *p* ≤ 0.05. Adobe Photoshop and Adobe Illustrator programs were used to compile images, graphs and text into final figures.

## Results

### Subject demographics

Demographic, clinical, and global neuropathological results for the 44 cases used in this study are summarized in Table 1. Notably, no significant differences were observed between the ND and MCI groups for age (*p* = 0.13), sex (*p* > 0.99), postmortem interval (PMI: mean = 2.7 + 0.6 h, range = 1.5–4.8 h, *p* = 0.84), Mini-Mental State Examination score (MMSE, *p* = 0.34), Braak stage (*p* = 0.85), NIA-Reagan AD probability level (*p* = 0.86) or CERAD plaque density (*p* = 0.96).

### Axonal tau pathology occurs in the absence of cell body pathology in the DG-mossy fiber pathway

**Fig. 1:**
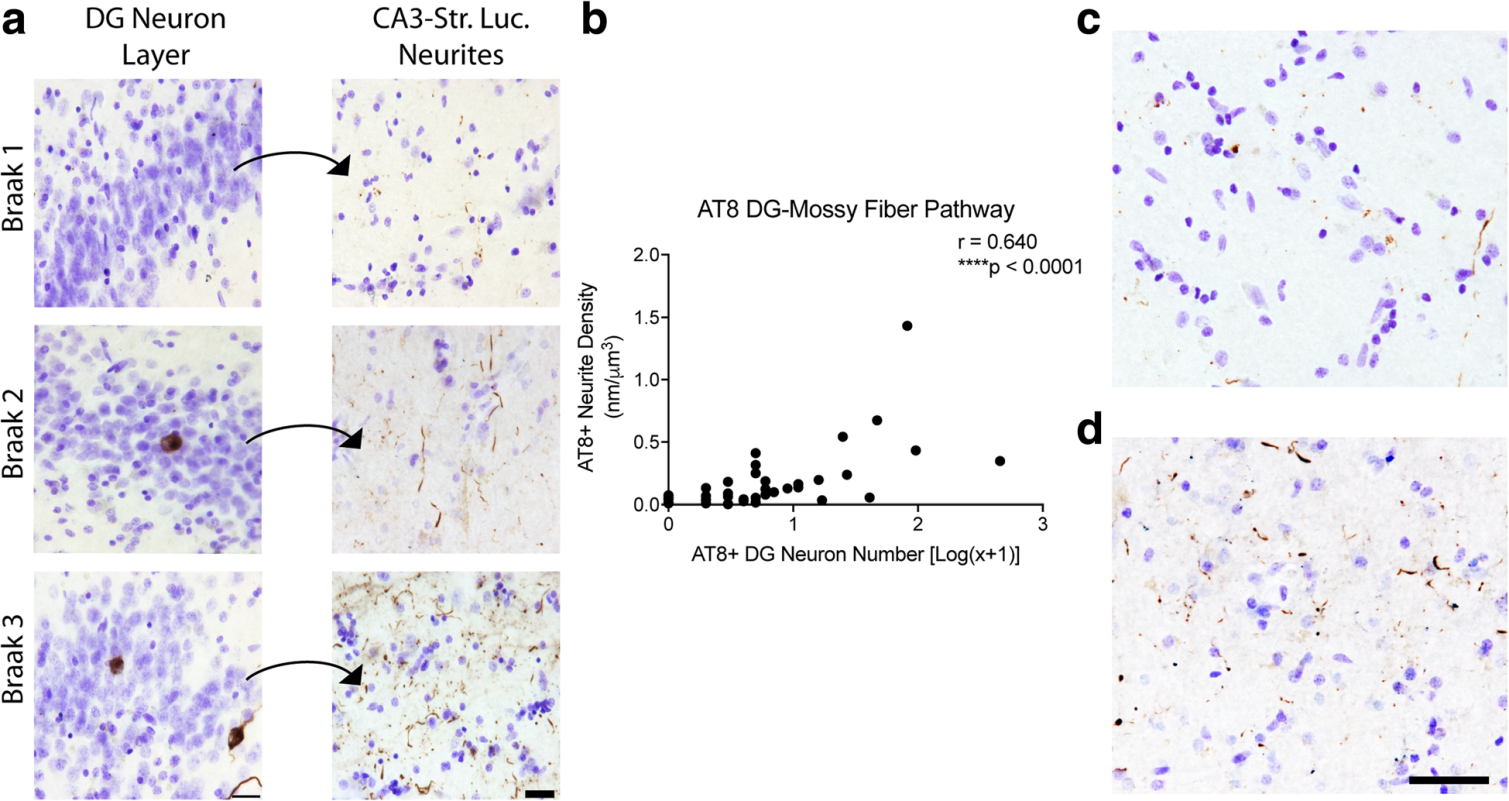
Axonal AT8 phosphorylation in the mossy fiber pathway occurs in the absence of DG cell body pathology. (**a**) AT8 staining in the dentate gyrus granule cell layer (DG) and their corresponding mossy fiber terminal fields in the stratum lucidum of CA3 (Str. Luc.) across Braak stages. All sections were counter stained with cresyl violet. The increase in cell body staining positively correlates with Braak staging (see Table 2). Scale bars are 25 μm. Quantification of cell body staining using total enumeration indicates the low number of positive cells in these cases. 71.4% of cases (30 of 42) displayed five or fewer cells stained in the DG, with 16.7% (7 of 42) showing no observable cell body pathology (see Table 3). By comparison, 100% of cases displayed axonal AT8 staining in the CA3 Str. Luc. (**b**). Spearman correlation analysis of AT8+ axonal density and cell body number in the mossy fiber pathway. Data points are plotted as each individual case. A strong, positive correlation (*r =* 0.640, *p* ≤ 0.0001) indicates an increase in axonal pathology as cell body pathology increases. (**c**-**d**). Representative images from cases with sparse (**c**) and dense (**d**) AT8+ mossy fiber axons in cases lacking cell body pathology demonstrate the extent of axonal pathology that can occur prior to observable somatodendritic pathology. Scale bars are 50 μm

We measured the amount of AT8+ immunoreactivity (Fig. 1) in the DG-mossy fiber pathway of the hippocampus. In this pathway, the granule cells (where cell body pathology was measured) give rise to axonal projections, known as mossy fibers, that terminate in the CA3 Str. Luc. (where neurites were measured). All cases displayed AT8+ neuropil threads in the CA3 Str. Luc., however, AT8+ staining in the corresponding cell bodies of the DG was less common (Fig. 1a). Specifically, 16.7% of cases (7 of 42) displayed no observable AT8+ staining in the DG cell bodies, but all of these cases showed AT8+ neurite staining in the CA3 Str. Luc. (Table 2). Axonal AT8 neurite density showed a significant positive correlation with the number of AT8+ cell bodies of the mossy fiber pathway (Spearman r = 0.640, *p* < 0.0001, Fig. 1b). In cases lacking AT8+ cell bodies in the DG, the observable mossy fiber pathology ranged from sparse (Fig. 1c) to moderate (Fig. 1d), and cases with cell body staining were never without neurite staining.

**Table 2:** Distribution of cases with different levels of local AT8 pathology in the dentate gyrus granule cells

AT8+ DG Cells	Number of Cases	Percent of Cases	Mean Neurite Density
0	7	16.7	0.043
1	5	11.9	0.056
2	5	11.9	0.084
3	4	9.5	0.034
4	5	11.9	0.209
5	4	9.5	0.129
≥6*	12	28.6	0.344

*DG* dentate gyrus; *full range was from 6 to 453 cells

**Fig. 2:**
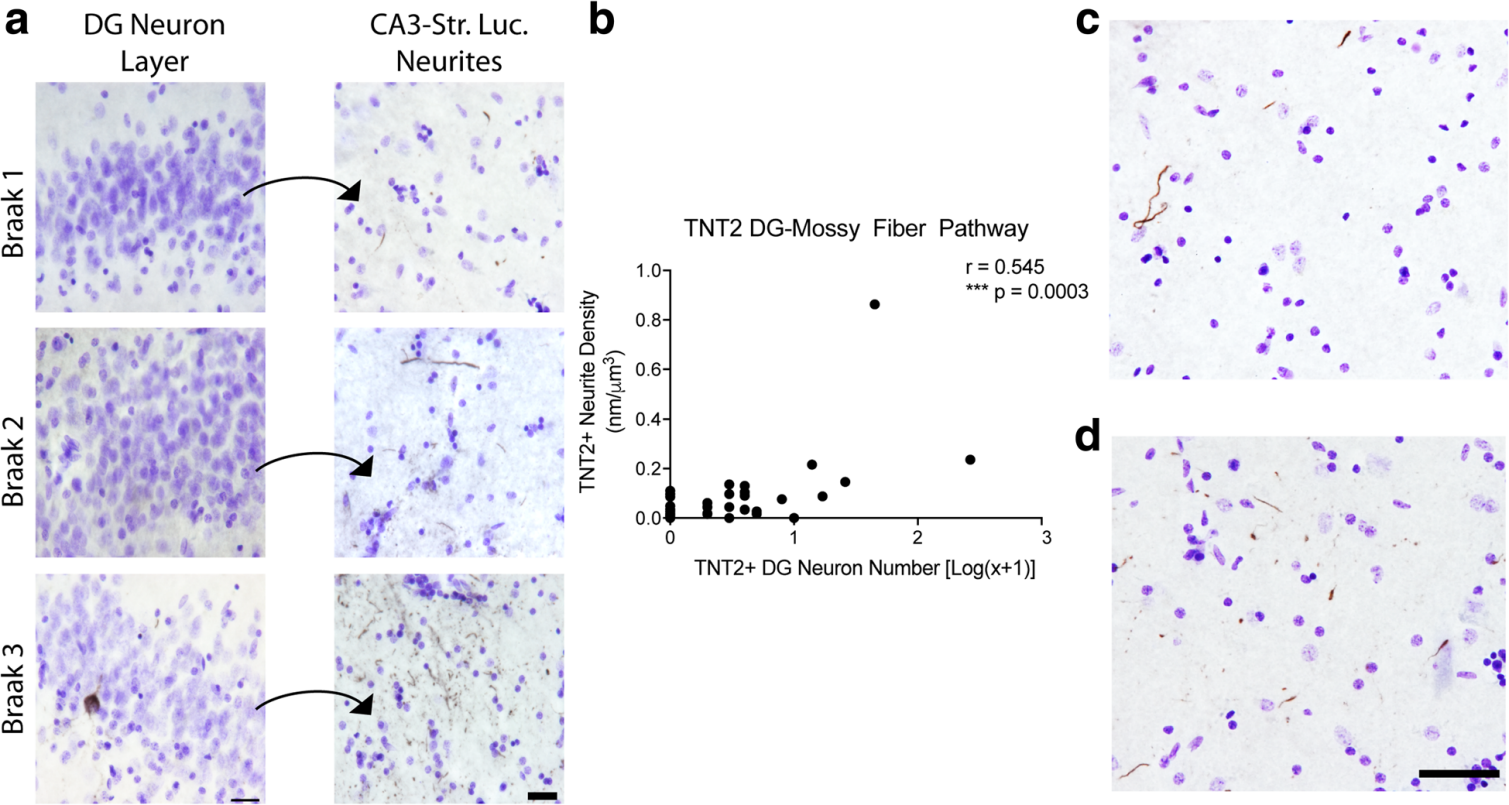
Axonal PAD exposure in the mossy fiber pathway occurs in the absence of DG cell body pathology. (**a**) TNT2 staining in the dentate gyrus granule cell layer (DG) and their corresponding mossy fiber terminal fields in the stratum lucidum of CA3 (Str. Luc.) across Braak stages. All sections were counter stained with cresyl violet. The increase in neuropil thread staining positively correlates with Braak staging (see Table 2). Scale bars are 25 μm. Quantification of cell body staining using total enumeration indicates the low number of positive cells in these cases. 82.1% of cases (32 of 39) displayed five or fewer cells stained in the DG, with 43.6% (17 of 39) showing no observable cell body pathology (see Table 5). A subset of cases without cell body staining still contained axonal TNT2 staining (see Table 5). (**b**). Spearman correlation analysis of TNT2+ axonal density and cell body number in the mossy fiber pathway. A strong, positive correlation (*r =* 0.545, *p* = 0.0003) indicates an increase in axonal pathology as cell body pathology increases. (c-d). Representative images from cases with sparse (**c**) and more dense (**d**) TNT2+ mossy fiber axons in cases lacking detectable cell body pathology demonstrate the extent of axonal pathology that can occur prior to observable somatodendritic pathology. Scale bars are 50 μm

Next, we analyzed the amount of TNT2+ immunoreactivity in the DG-mossy fiber pathway (Fig. 2 and Table 3) as a measure of PAD-exposed tau, an early pathological event in tauopathies [18]. The majority of cases displayed TNT2+ staining in the CA3 Str. Luc. (83%; 34 of 41; Fig. 2a). Additionally, 17 cases (43.6%) contained no observable TNT2+ DG neurons, and among these cases 10 contained TNT2+ neurite pathology in the CA3 Str. Luc. mossy fibers and 7 did not contain TNT2+ neurites. In contrast to AT8 pathology, there were cases that contained no observable TNT2+ pathology in the DG cell bodies or CA3 Str. Luc. layer (17%; 7 of 41). Axonal TNT2+ neurite staining in the mossy fiber pathway displayed a significant positive correlation with the number of TNT2+ DG cell bodies (Spearman r = 0.702, *p* ≤ 0.0001, Fig. 2b). In cases lacking TNT2+ cell bodies in the DG, the observable mossy fiber pathology ranged from sparse (Fig. 2c) to moderate (Fig. 2d), and cases with cell body staining were never without neurite staining.

**Table 3:** Distribution of cases with different levels of local TNT2 pathology in DG granule cells

TNT2+ DG Cells	Number of Cases	Percent of Cases	Mean Neurite Density
0	17	43.6	0.027
1	4	10.3	0.035
2	4	10.3	0.070
3	5	12.8	0.079
4	2	5.1	0.022
5	0	0	0
≥6*	7	17.9	0.232

*DG* dentate gyrus, *full range was from 6 to 265 cells

The local AT8+ or TNT2+ neurite and cell densities in the DG-mossy fiber pathway were correlated with demographic or global neuropathology scores (Table 4). Local AT8+ and TNT2+ neurite densities showed a significant positive correlation with age (AT8+ and TNT2+, NIA-Reagan (AT8+ only), global tangle density (TNT2+ only) and Braak Stage (TNT2+ only) in the mossy fiber pathway. Local AT8+ and TNT+ neurite densities in the mossy fiber pathway were not correlated with MMSE or PMI (data not shown). In the DG cell layer, AT8+ cell number showed a significant positive correlation with Braak stage and global tangle density, while TNT2+ cell number did not correlate with any measure. Finally, no statistical differences were observed between AT8+ or TNT2+ neurite density in the CA3 Str. Luc. when compared by either diagnosis states (ND or MCI) or sex groups (Additional file 4: Figure S4).

**Table 4:** Spearman correlations between demographic, cognitive, or global neuropathological measures and tau markers in local axonal and somatodendritic compartments of the DG-mossy fiber pathway

Region	Tau Marker	Age	Braak Stage	Global CERADPlaque Density	Global Tangle Density	NIA-Reagan Score
DG Granule Cell Density	**AT8**	r = 0.299 *p* = 0.058	r = 0.472* ***p = 0.002***	r = 0.069 *p* = 0.670	r = 0.504* ***p*** **= 0.0008**	r = 0.394 *p* = 0.01
	**TNT2**	r = 0.255 *p* = 0.113	r = 0.280 *p* = 0.080	r = 0.004 *p* = 0.702	r = 0.239 *p* = 0.137	r = 0.253 *p* = 0.116
Mossy Fiber Density	**AT8**	r = 0.446* ***p*** **= 0.004**	r = 0.337 *p* = 0.036	r = 0.205 *p* = 0.211	**r = 0.418** ***p*** **= 0.008**	r = 0.470* ***p*** **= 0.003**
	**TNT2**	r = 0.477* ***p = 0.002***	r = 0.484* ***p = 0.002***	r = 0.156 *p* = 0.344	r = 0.539* ***p = 0.0004***	**r = 0.420** ***p = 0.008***

*PMI* post-mortem interval, *MMSE* mini mental state exam, *NIA-Reagan* National Institute on Aging-Reagan Institute AD probability level, *CA* cornu ammonis, *DG* dentate gyrus; Braak stages ranged from 1 to 3. * indicates a significant correlation (*p* ≤ 0.005, adjusted *p*-value for multiple comparisons)

### Axonal tau pathology occurs without cell body pathology in the CA3-Schaffer collateral pathway

**Fig. 3:**
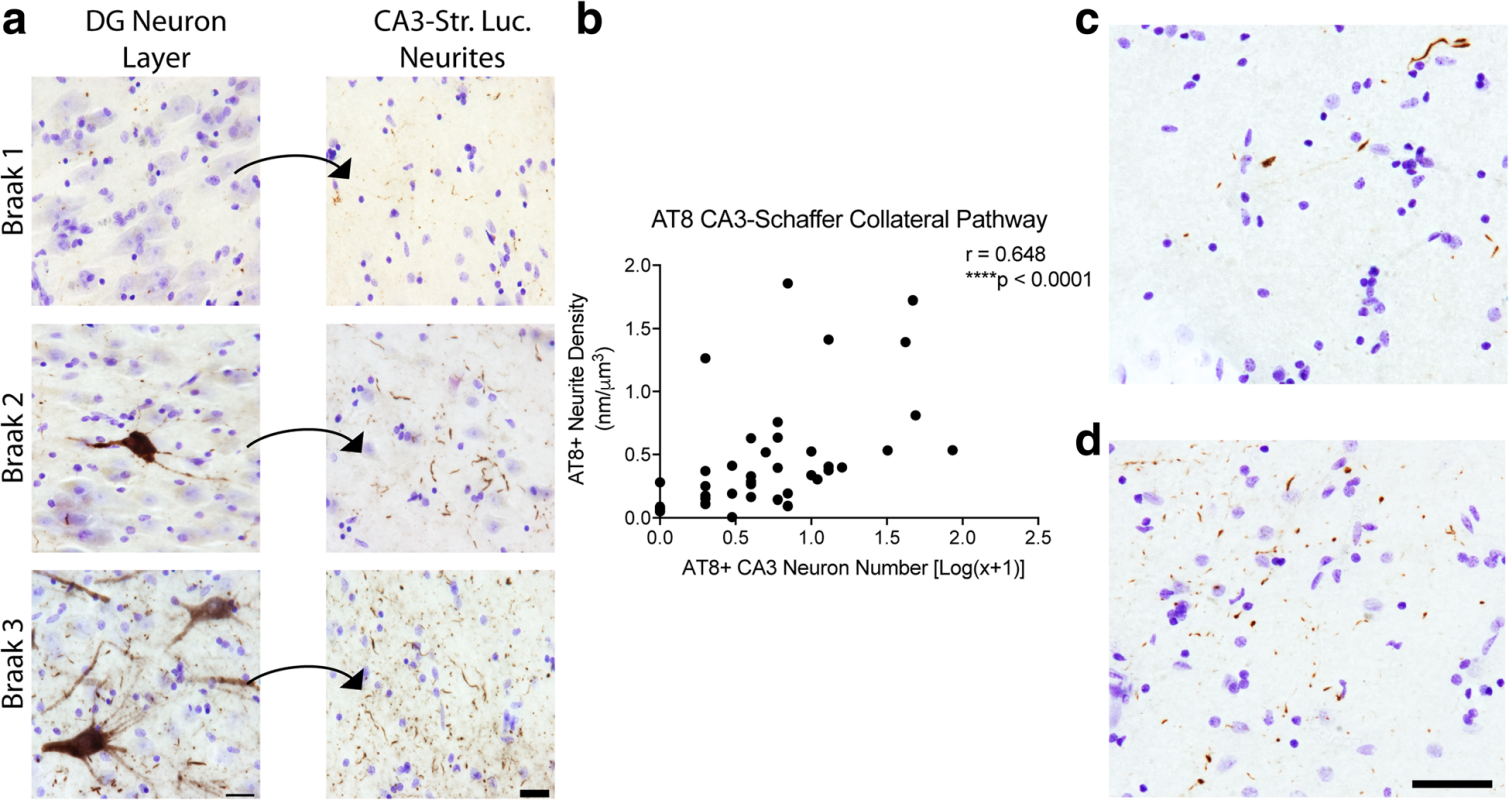
Axonal AT8 phosphorylation in the Schaffer collateral pathway occurs in the absence of CA3 cell body pathology. (**a**). AT8 staining in the pyramidal cell layer of CA3 and their corresponding Schaffer collateral terminal fields in the stratum radiatum of CA1 (Str. Rad.) across Braak stages. All sections were counter stained with cresyl violet. The increase in cell body staining and neuropil thread staining positively correlates with Braak staging (see Table 2). Scale bars are 25 μm. Quantification of cell body staining using total enumeration indicates the low number of cells stained in these cases. 61.5% of cases (24 of 39) displayed five or fewer cells stained in the CA3 pyramidal cell layer, with 12.8% (5 of 39) showing no observable cell body pathology (see Table 4). By comparison, 100% of cases displayed axonal AT8 staining in the CA1 Str. Rad. (**b**). Spearman correlation analysis of AT8+ axonal density and cell body number in the Schaffer collateral pathway. A strong, positive correlation (*r =* 0.648, *p* ≤ 0.0001) indicates an increase in axonal pathology as cell body pathology increases. (**c**-**d**). Representative images from cases with sparse (**c**) and dense (**d**) AT8+ Schaffer collateral axons in cases lacking cell body pathology demonstrate the extent of axonal pathology that can occur prior to observable somatodendritic pathology. Scale bars are 50 μm

We measured the amount of AT8+ immunoreactivity (Fig. 3) in the CA3-Schaffer collateral pathway of the hippocampus. In this pathway, the CA3 pyramidal neurons (where cell body pathology was measured) give rise to axonal projections, known as Schaffer collaterals, that terminate in the CA1 Str. Rad. (where neurites were measured). All cases displayed AT8+ neuropil threads within the CA1 Str. Rad., however, AT8+ cell bodies within the CA3 pyramidal layer were less common (Fig. 3a). All cases showed AT8+ neurite staining in the CA1 Str. Rad. region, but in 12.8% of cases (5 of 39) there was no observable AT8+ cell bodies in the CA3 pyramidal layer (Table 5). Axonal AT8 neurite staining displayed a significant positive correlation with the number of AT8+ cell bodies of the Schaffer collateral pathway (Spearman r = 0.648, *p* ≤ 0.0001, Fig. 3b). Importantly, in the absence of observable cell body pathology, the terminal region contained a range of tau pathology from relatively sparse (Fig. 3c) to relatively dense neurite staining (Fig. 3d), and cases with cell body staining were never without neurite staining.

**Table 5:** Distribution of cases with different levels of local AT8 pathology in CA3 pyramidal cells

AT8+ CA3 Cells	Number of Cases	Percent of Cases	Mean Neurite Density
0	5	12.8	0.114
1	6	15.4	0.386
2	3	7.7	0.202
3	5	12.8	0.333
4	1	2.6	0.518
5	4	10.3	0.482
≥6*	15	38.5	0.726

*CA* cornu ammonis; *full range was from 6 to 85

**Fig. 4:**
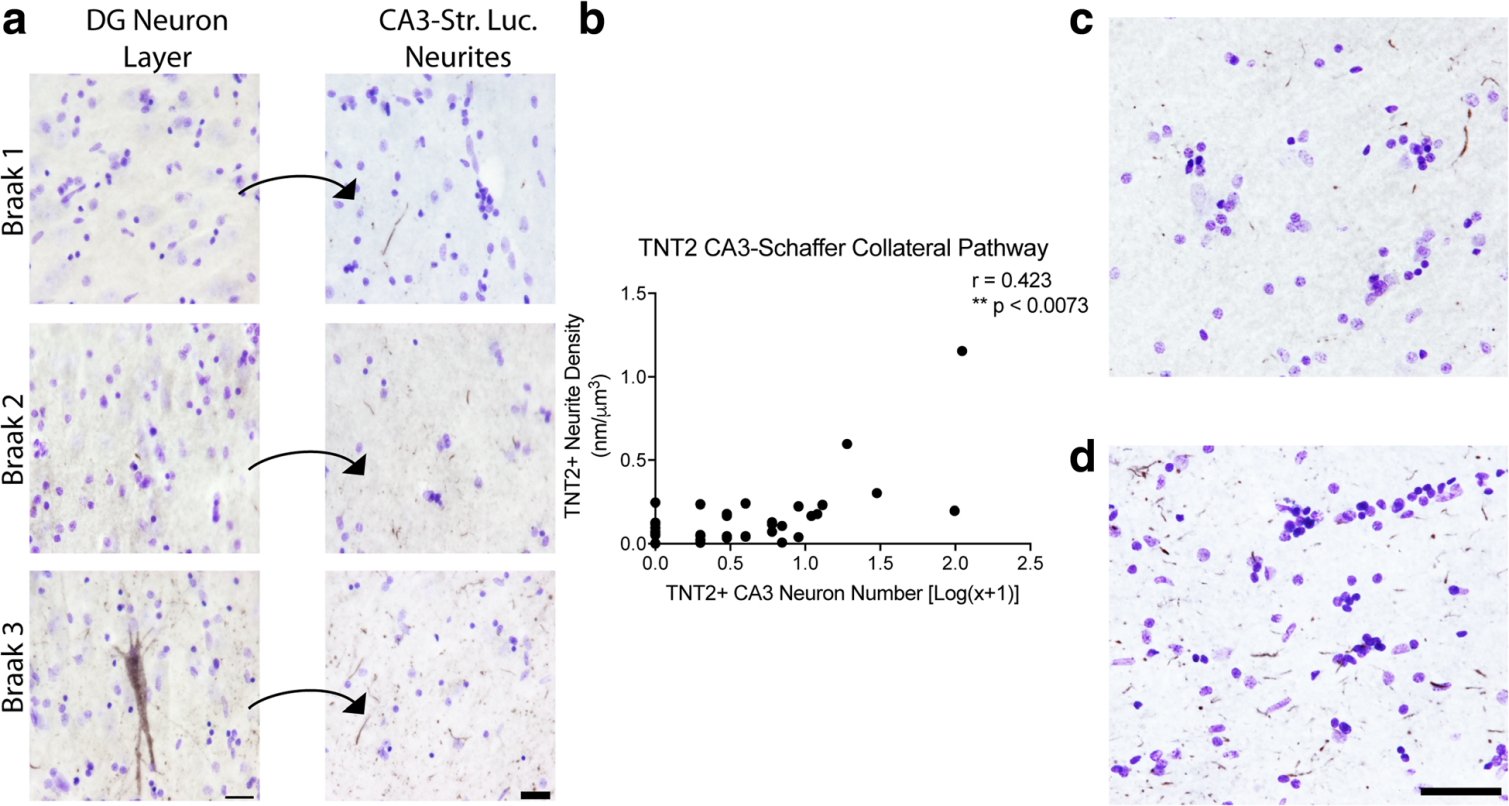
Axonal PAD exposure in the Schaffer collateral pathway occurs in the absence of CA3 cell body pathology. (**a**) TNT2 staining in the pyramidal cell layer of CA3 pyramidal cell layer and their corresponding mossy fiber terminal fields in the CA1 stratum radiatum (Str. Rad.) across Braak stages. All sections were counter stained with cresyl violet. The increase in neuropil thread staining positively correlates with Braak staging (see Table 6). Scale bars are 25 μm. Quantification of cell body staining using total enumeration indicates the low number of positive cells in these cases. 71.8% of cases (28 of 39) displayed five or fewer cells stained in the CA3 pyramidal cell layer, with 25.6% (10 of 39) showing no observable cell body pathology (see Table 6). A subset of cases without cell body staining still contained axonal TNT2 staining (see Table 6). (**b**). Spearman correlation analysis of TNT2+ axonal density and cell body number in the mossy fiber pathway. A moderate, positive correlation (*r =* 0.423, *p* = 0.0073) indicates an increase in axonal pathology as cell body pathology increases. (**c**-**d**). Representative images from cases with sparse (**c**) and dense (**d**) TNT2+ Schaffer collateral axons in cases lacking cell body pathology demonstrate the extent of axonal pathology that can occur prior to observable somatodendritic pathology. Scale bars are 50 μm

Next, we analyzed the amount of TNT2+ immunoreactivity in the CA3-Schaffer collateral pathway (Fig. 4) as a measure of PAD-exposed tau [18]. All but one case displayed TNT2+ neurite staining in the CA1 Str. Rad. (Fig. 4a). Specifically, 25.6% of cases (10 of 39) displayed TNT2+ neurite staining in the CA1 Str. Rad., but no observable TNT2+ staining in the CA3 pyramidal cell body layer (Table 6). Axonal TNT2+ neurite staining in the Schaffer collaterals showed a significant positive correlation with the number of TNT2+ CA3 cell bodies (Spearman r = 0.719, *p* ≤ 0.0001, Fig. 4b). In cases displaying no TNT2+ cell bodies, the TNT2+ neurite pathology in the terminal region of this pathway contained pathology ranging from relatively sparse (Fig. 4c) to relatively dense (Fig. 4d). Cases with TNT2+ cell bodies staining were never without neurite staining.

**Table 6:** Distribution of cases with different levels of local TNT2 pathology in CA3 pyramidal cells

TNT2+ CA3 Cells	Number of Cases	Percent of Cases	Mean Neurite Density
0	10	25.6	0.083
1	6	15.4	0.069
2	4	10.3	0.107
3	4	10.3	0.092
4	0	0	0
5	4	10.3	0.111
≥6*	11	28.2	0.291

*CA* cornu ammonis, *full upper range was from 6 to 110 cells

Finally, the local AT8+ or TNT2+ neurite and cell densities in the CA3-Schaffer collateral pathway were correlated with demographic or global neuropathology scores (Table 7). Local AT8+ and TNT2+ neurite densities showed a significant positive correlation with Braak stage, NIA-Reagan, and global tangle density in the Schaffer collateral pathway, while AT8+ neurites were correlated with age but TNT2+ neurites were not. Local AT8+ and TNT+ neurite densities in the Schaffer collateral pathway were not correlated with MMSE or PMI (data not shown). In the CA3 pyramidal cell layer, AT8+ cell number showed a significant positive correlation with Braak stage, global tangle density and NIA-Reagan score, while TNT2+ cell number did not correlate with any measure. No statistical differences were observed between AT8+ or TNT2+ neurite density in the CA1 Str. Rad. when compared to either diagnosis states (ND or MCI) or sex (Additional file 5: Figure S5).

**Table 7:** Spearman correlations between demographic, cognitive, or global neuropathological measures and tau markers in local axonal and somatodendritic compartments of the CA3-Schaffer collateral pathway

Region	Tau Marker	Age	Braak Stage	Global CERADPlaque Density	Global Tangle Density	NIA-Reagan Score
CA3 Pyramidal Cell Density	**AT8**	r = 0.344 *p* = 0.032	r = 0.462* ***p*** ** = 0.003**	r = 0.241 *p* = 0.139	r = 0.441* ***p*** **= 0.005**	r = 0.508* ***p*** ** = 0.001**
	**TNT2**	r = 0.227 *p* = 0.165	r = 0.213 *p* = 0.193	r = −0.062 *p* = 0.979	r = 0.322 *p* = 0.046	r = 0.135 *p* = 0.412
Schaffer Collateral Fiber Density	**AT8**	r = 0.521* ***p*** ** = 0.0003**	r = 0.524* ***p*** **= 0.0003**	r = 0.109 *p* = 0.481	r = 0.533* ***p*** **= 0.0002**	r = 0.456* ***p*** ** = 0.002**
	**TNT2**	r = 0.305 *p* = 0.044	r = 0.497* ***p*** **= 0.0006**	r = 0.331 *p* = 0.028	r = 0.522* ***p*** ** = 0.0003**	r = 0.567* ***p*** ** = 0.0006**

*PMI* post-mortem interval, *MMSE* mini mental state exam, *NIA-Reagan* National Institute on Aging-Reagan Institute AD probability level, *CA* cornu ammonis, *DG* dentate gyrus, Braak stages ranged from 1 to 3. * indicates a significant correlation (*p* ≤ 0.005, adjusted p-value for multiple comparisons)

### Early AT8 and TNT2 pathology in the stratum lucidum and stratum radiatum colocalizes with axons

**Fig. 5:**
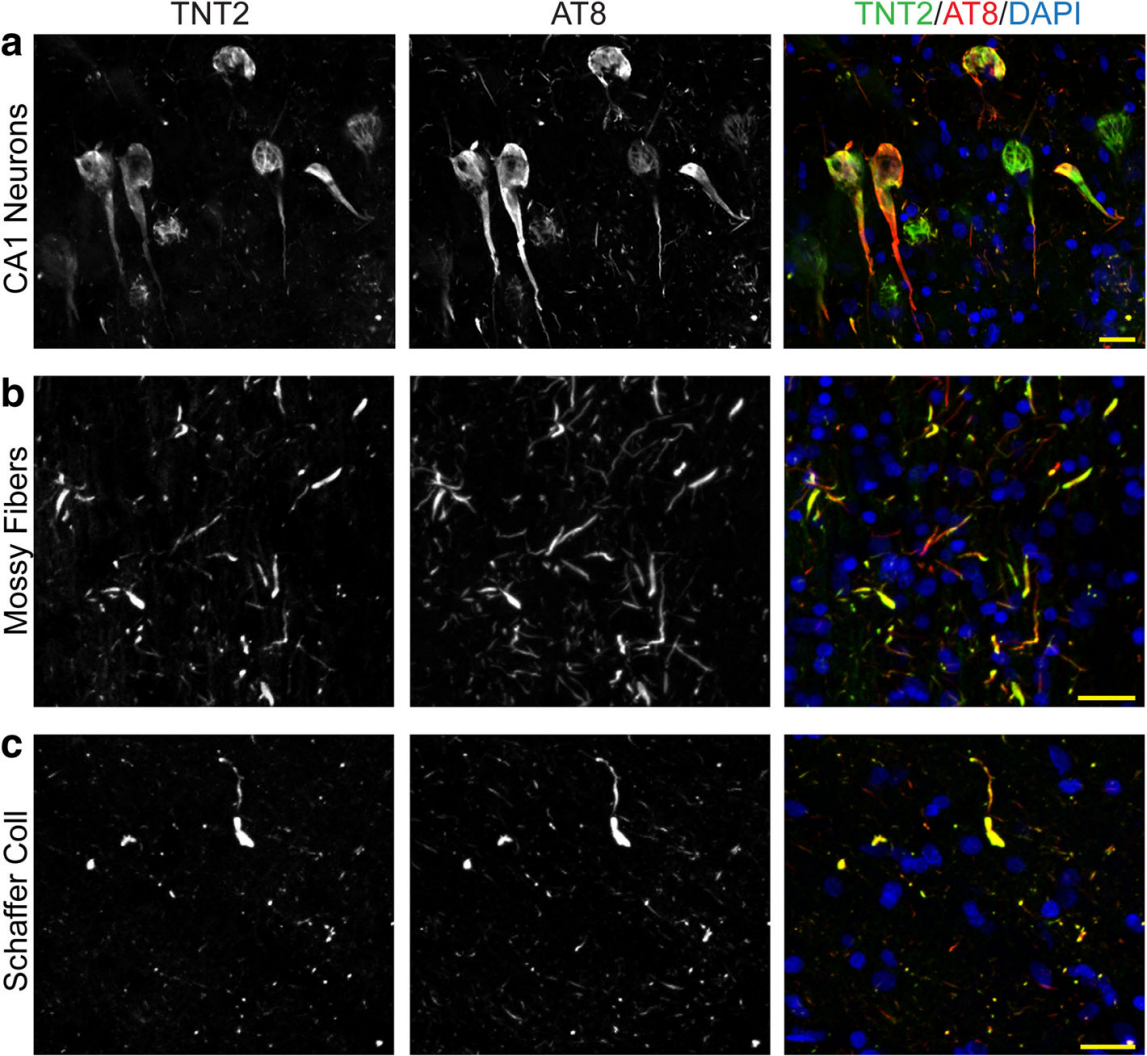
AT8+ and TNT2+ tau pathologies are highly colocalized. Hippocampal sections were stained with AT8 and TNT2 tau antibodies. (**a**) Both AT8 and TNT2 are highly colocalized within hippocampal pyramidal neuronal inclusions (CA1 region depicted) as previously demonstrated [41]. (**b**) Neurites within the CA3 Str. Luc., the axonal region for mossy fibers, showed a high level of colocalization with AT8 and TNT2 tau antibodies. (**c**) Similarly, neurites in the CA1 Str. Rad., the axonal region for Schaffer collaterals, demonstrated extensive colocalization between AT8 and TNT2. Scale bars are 25 μm

To confirm that the AT8+ and TNT2+ pathologies colocalized in the current tissue cohort we used multi-label immunofluorescence. As previously observed [41], extensive colocalization was observed between AT8 and TNT2 in hippocampal pyramidal neurons (Fig. 5). Here, we demonstrate that extensive colocalization occurs between AT8 and TNT2 in both CA3-mossy fiber and CA1-Schaffer collateral neurites within the hippocampus. Primary delete control sections where one tau antibody was omitted produced the expected pattern of staining confirming the specificity of each label (Additional file 2: Figure S2).

**Fig. 6:**
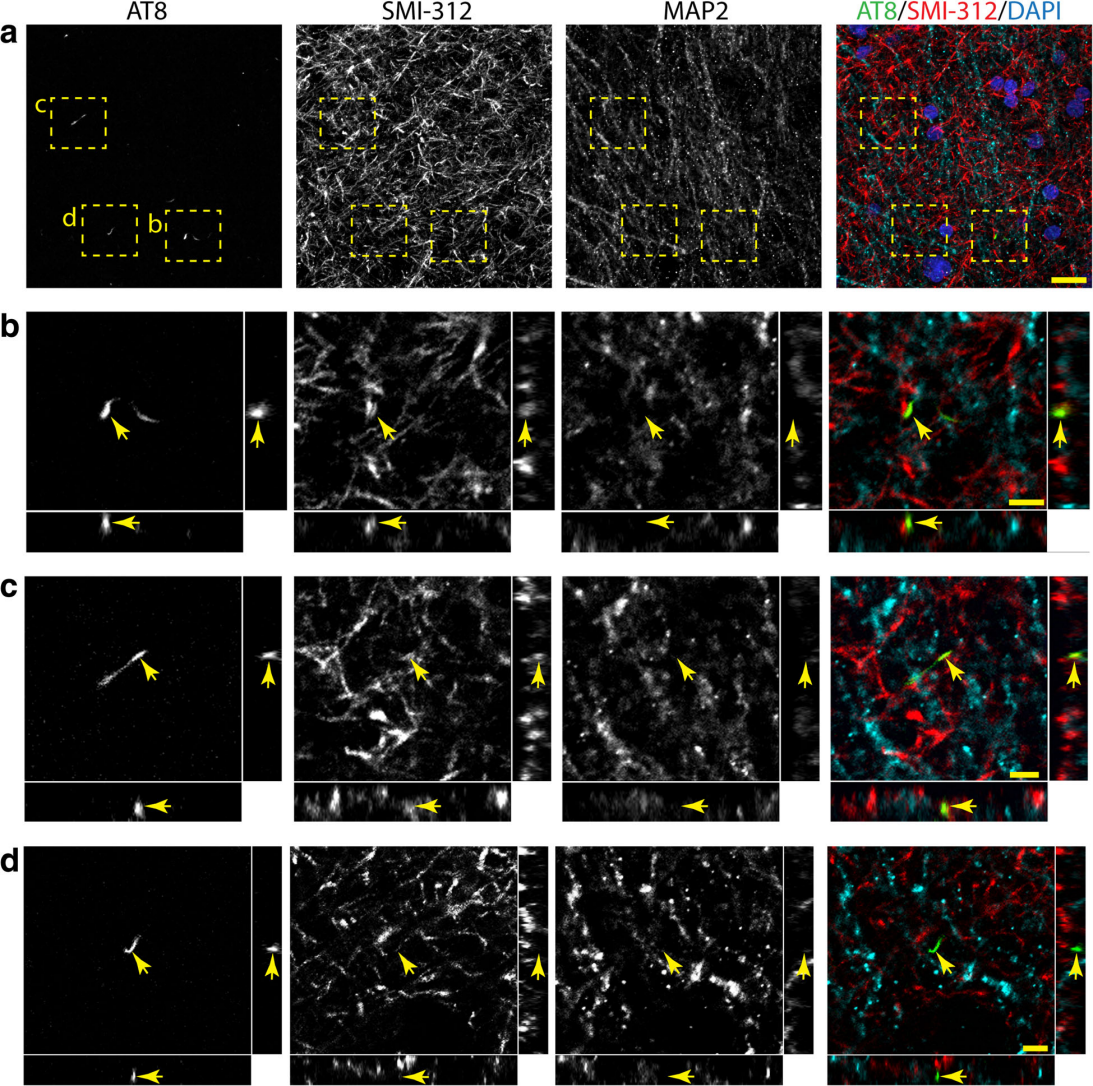
AT8+ tau neurite pathology colocalizes with the axonal marker SMI-312 in the mossy fiber pathway. (**a**-**d**). Representative images of AT8 (green), SMI-312 (red) and MAP2 (cyan) triple labeling immunofluorescence staining in the mossy fibers of the hippocampus (merged image in a includes DAPI nuclear counter stain). (**a**) A low magnification image shows axonal (SMI-312), dendritic (MAP2) and tau+ neurites (AT8) in the CA3 Str. Luc. region of the hippocampus in a Braak stage III case. (**b** and **c**) Crossectional analysis of z-stack images (60x magnification, 0.5 μm step size) demonstrate that AT8+ neurites colocalize with SMI-312 (arrows). (**d**) Cross-sectional analysis of z-stack images show that some AT8+ neurites do not colocalize with SMI-312 or MAP2. Notably, little to no colocalization was observed between AT8+ neurites and MAP2. Scale bars are 20 μm for a and 5 μm for b-d. TNT2+ tau neurite pathology showed similar colocalization with SMI-312, not MAP2 in the mossy fiber pathway (see Additional file 6: Figure S6)

**Fig. 7:**
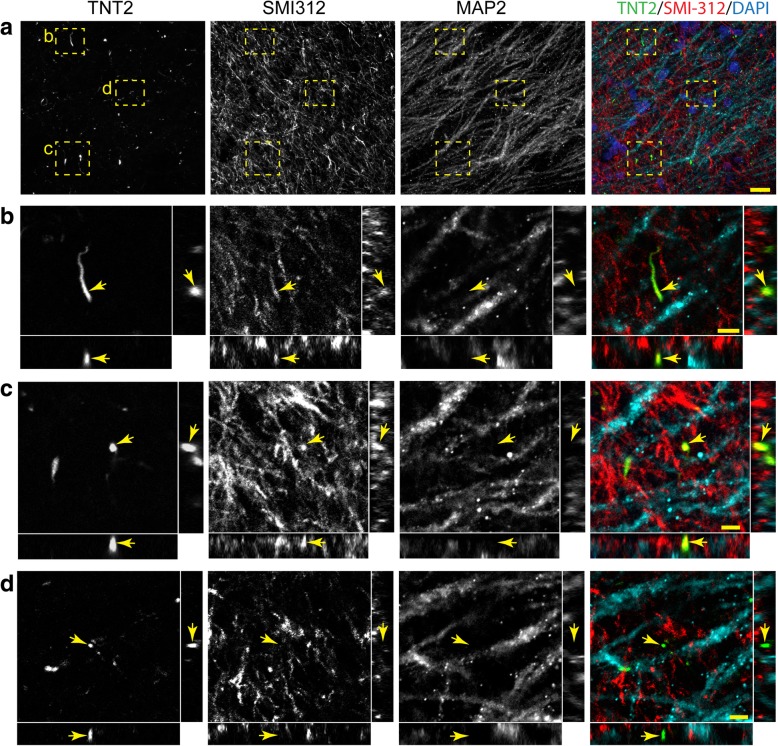
TNT2+ tau neurite pathology colocalizes with the axonal marker SMI-312 in the Schaffer collateral pathway. (**a**-**d**). Representative images of TNT2 (green), SMI-312 (red) and MAP2 (cyan) triple labeling immunofluorescence staining in the Schaffer collaterals of the hippocampus (merged image in a includes DAPI nuclear counter stain). (**a**) A low magnification image shows axonal (SMI-312), dendritic (MAP2) and tau+ neurites (TNT2) in the CA1 Str. Rad. region of the hippocampus in a Braak stage III case. (**b** and **c**) Cross-sectional analysis of z-stack images (60x magnification 0.5 μm step size) demonstrate that TNT2+ neurites colocalize with SMI-312 (arrows). (**d**) Cross-sectional analysis of z-stack images show that some TNT2+ neurites do not colocalize with SMI-312 or MAP2. Notably, little to no colocalization was observed between TNT2+ neurites and MAP2. Scale bars are 20 μm for a and 5 μm for b-d. AT8+ tau neurite pathology showed similar colocalization with SMI-312, not MAP2 in the Schaffer collateral pathway (see Additional file 6: Figure S6)

Next, we used multi-label immunofluorescence to determine whether AT8 and TNT2 pathologies in the DG-mossy fiber and CA3-Schaffer collateral pathway were axonal (i.e. SMI-312+ neurites) or dendritic (i.e. MAP2+ neurites). The colocalization between these markers was performed using high magnification z-stacks that were taken in the CA3 Str. Luc (i.e. mossy fibers) and CA1 Str. Rad (i.e. Schaffer collaterals). In the CA3 Str. Luc. region (mossy fibers), AT8+ (Fig. 6) and TNT2+ (Additional file 6: Figure S6a-b) neurites showed moderate colocalization with SMI-312, an axonal marker. Similarly, AT8+ (Additional file 6: Figure S6c-d) and TNT2+ (Fig. 7) neurites showed moderate colocalization with SMI312, in the CA1 Str. Rad. region (Schaffer collaterals). In contrast, little to no colocalization was observed between AT8 or TNT2 and MAP2+ neurites in the images analyzed within the CA3 Str. Luc. and CA1 Str. Rad. regions. It is noteworthy that numerous AT8+ (Figs. 6d) and TNT2+ (Figs. 7d) neurites did not colocalize with either SMI-312 or MAP2.

### Early axonal AT8+ and TNT2+ tau pathology is independent of amyloid-β pathology in the DG-mossy fiber and CA3-Schaffer collateral pathways

**Fig. 8:**
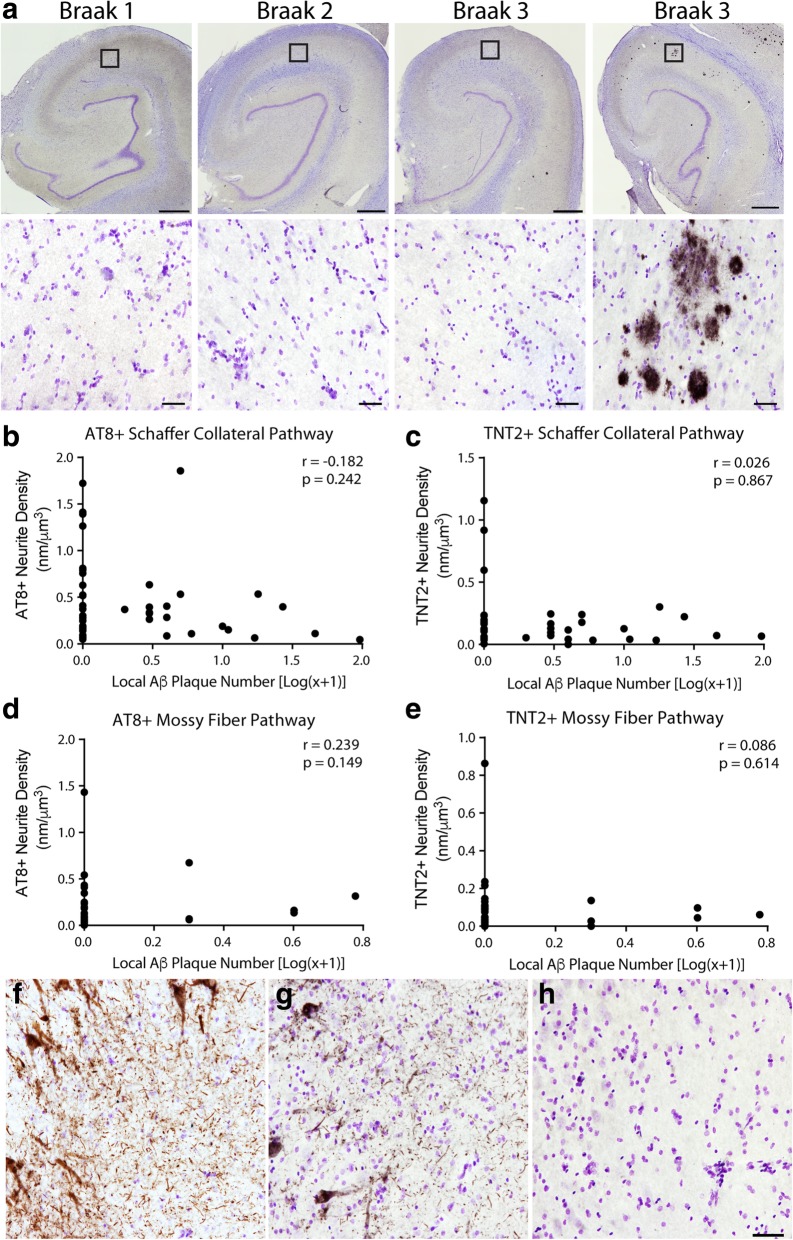
Local tau pathology is observed in the absence of local amyloid-β pathologies. (**a**). Representative hippocampi across Braak stages stained with MOAB2. Note the rarity of MOAB2+ plaques in the hippocampus and in the Schaffer collateral terminal region of the CA1 Str. Rad. (similar lack of Aβ pathology occurred in the mossy fiber terminal region of the Str. Luc.). A representative image of the relatively rare cases containing a significant amyloid load in the hippocampus is shown to confirm the effective labeling of Aβ plaques with MOAB2 antibody. Boxes indicate the location of higher magnification images. In the mossy fiber pathway, 84.2% of cases contained zero plaques in the CA3 Str. Luc. and 59% of cases contained zero plaques in the DG (see Table 7). In, the Schaffer collateral pathway, 55.8% of cases contained zero plaques in the CA1 Str. Rad. and 78.9% of cases contained zero plaques in the CA3 pyramidal cell layer (see Table 7). Scale bars are 1 mm in upper images and 50 μm in lower images. (**b**, **c**). No significant correlation was observed in the Schaffer collateral pathway between the presence of local Aβ plaques and local AT8+ staining (**b**, Spearman r = − 0.182, *p* = 0.242) or TNT2+ staining (**c**, Spearman r = 0.026, *p* = 0.867). (**d**, **e**). No significant correlation was observed in the mossy fiber pathway between the presence of local Aβ plaques and local AT8+ staining (**d**, Spearman r = 0.239, *p* = 0.149) or TNT2+ staining (**e**, Spearman r = 0.086, *p* = 0.614). (**f**, **h**). Representative images of AT8 (**f**), TNT2 (**g**), and MOAB2 (**h**) staining in the same location within the CA3 region from the same case illustrates the robust tau pathology possible in the absence of detectable Aβ pathology. It is noteworthy that no cases contained intraneuronal MOAB-2 staining for soluble monomeric or oligomeric Aβ species. The only Aβ pathologies observed in these hippocampal regions were extracellular plaques. Scale bar is 50 μm and applies to panels **f**, **g**, and **h**

Finally, we stained for Aβ plaques in the CA3-Schaffer collateral and DG-mossy fiber pathway regions using the MOAB2 antibody [77] and counted local plaques using total enumeration (Fig. 8a). The majority of cases did not contain detectable MOAB-2+ Aβ pathology in the local regions of the hippocampal formation assessed (i.e. DG cell layer, CA3 Str. Luc, CA3 cell layer, and CA1 Str. Rad.). Specifically, 84.2% contained zero plaques in the CA3 Str. Luc. and 59% contained zero plaques in the DG, while 55.8% had zero plaques in the CA1 Str. Rad. and 78.9% contained zero plaques in the CA3 pyramidal cell layer (Table 8). Intracellular Aβ pathologies (e.g. monomeric or soluble oligomeric Aβ species) were not observed in the DG or CA3 neurons of any case used in this study.

**Table 8:** Distribution of cases with different levels of local Aβ pathology in the mossy fiber and Schaffer collateral pathway regions

Local Aβ Plaques	% Cases (N) (CA1 Str. Rad.)	% Cases (N) (CA3 Layer)	% Cases (N) (CA3 Str. Luc.)	% Cases (N) (DG Layer)
0	55.8 (24)	78.9 (30)	84.2 (32)	59.0 (23)
1	2.3 (1)	7.9 (3)	7.9 (3)	7.7 (3)
2	11.6 (5)	5.3 (2)	0 (0)	12.8 (5)
3	7.0 (3)	2.6 (1)	5.3 (2)	10.3 (4)
4	4.7 (2)	2.6 (1)	0 (0)	2.6 (1)
≥5*	18.6 (8)	2.6 (1)	2.6 (1)	7.7 (3)

*Aβ* amyloid-β, *Str.Rad* Stratum radiatum, *CA* cornu ammonis, *Str. Luc* stratum lucidum, *DG* dentate granule, *full range was from 5 to 13 plaques

Interestingly, neither AT8 phosphorylation nor PAD exposed tau correlated with the presence of local Aβ plaques in both the Schaffer collateral (Spearman r = − 0.182, *p* = 0.242 for AT8, and r = 0.026, *p* = 0.867 for TNT2, Fig. 8b, c) and mossy fiber pathways (Spearman r = 0.293, *p* = 0.149 for AT8, and r = 0.086, *p* = 0.614 for TNT2, Fig. 8d, e). The lack of correlation between AT8+ or TNT2+ neurite pathology in these regions remained even when cases with no global Aβ pathology (i.e. non-PART) were excluded (data not shown), with exception of a significant negative correlation between AT8+ neurite density and local plaque number in the Schaffer collaterals (Spearman r = − 0.379, *p* = 0.039). The extent of both AT8+ and TNT2+ tau pathology in some cases was remarkably robust in regions devoid of observable Aβ plaques (Fig. 8f-h). Importantly, the cases used here display the expected ranges of global Aβ pathologies consistent with aged ND and MCI cases, with the vast majority of cases displaying some degree of global Aβ pathology accumulation (31 of 44 cases; see Tables 1, 4 and 7). Taken together, these results indicate that AT8 phosphorylation and PAD exposure occur independently of the presence of Aβ plaque pathology in the Schaffer collateral and mossy fiber pathways of the hippocampus.

### Mossy fiber and Schaffer collateral pathway tau pathology in PART and non-PART cases

**Fig. 9:**
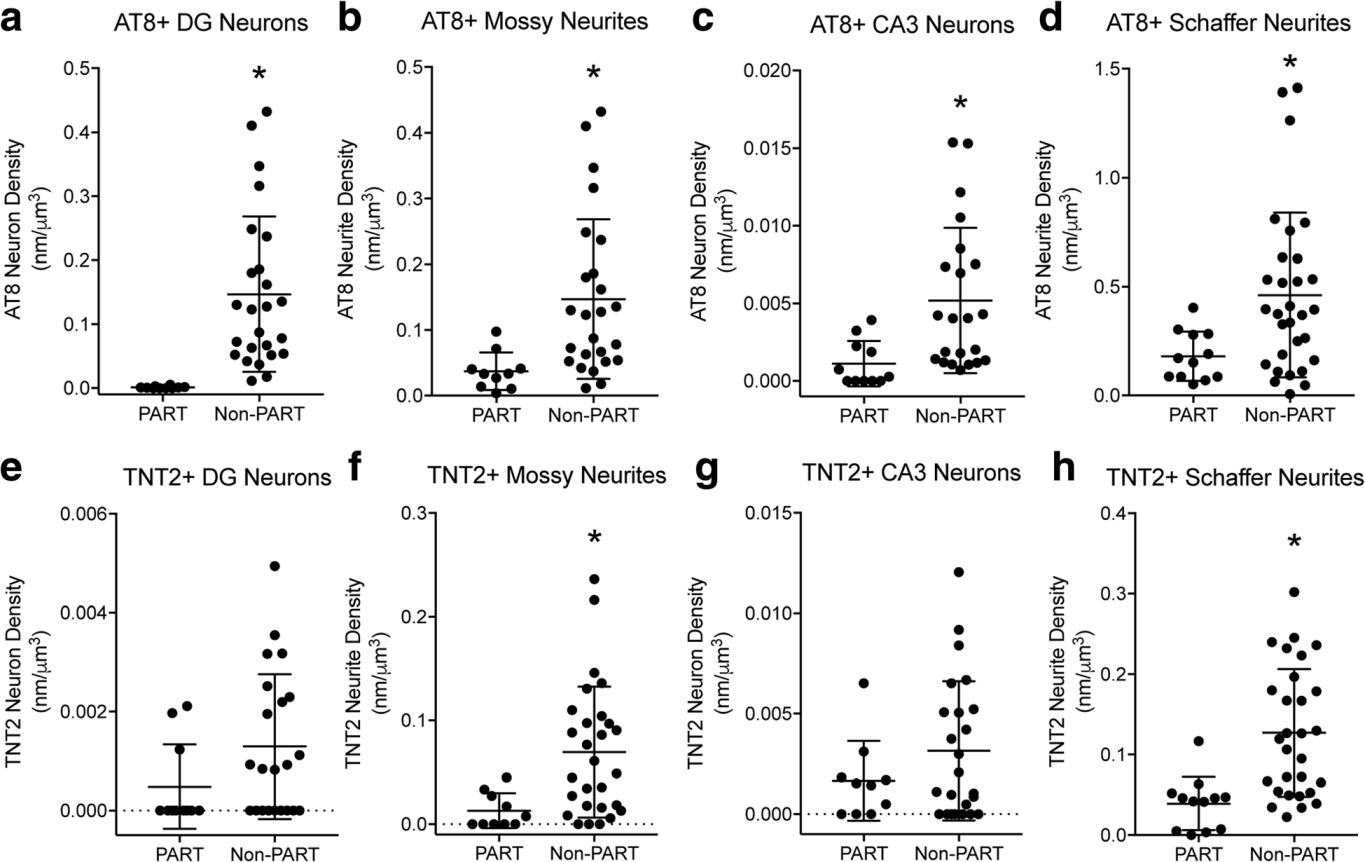
Non-PART cases contained greater levels of AT8+ and TNT2+ neurites and neurons in the DG-mossy fiber and CA3-Schaffer collateral pathways. (**a**-**b**) AT8+ neurons (**a**; *p* < 0.0001) and neurites (**b**; *p* = 0.0117) were significantly increased in non-PART cases compared to PART cases in the DG-mossy fiber pathway. (c-d) In the CA3-Schaffer collateral pathway, AT8+ neurons (**c**; *p* = 0.0024) and neurites (**d**; *p* = 0.0315) were significantly increased in non-PART cases compared to PART cases. (**e**-**f**) TNT2+ mossy neurites (**f**; *p* = 0.0175) were significantly increased in non-PART cases compared to PART cases, but not TNT2+ DG neurons (**e**; *p* = 0.0626) in the DG-mossy fiber pathway. (**g**-**h**) In the CA3-Schaffer collateral pathway, TNT2+ neurites (**h**; *p* = 0.0011) were significantly increased in non-PART cases compared to PART cases, but not TNT2+ CA3 neurons (**g**; *p* = 0.4232). All comparisons made using Mann-Whitney U-test, the data are median with interquartile range, and split y-axis were used with some data sets to better illustrate data spread

Recent work described PART where brains contain AD-like NFT pathology, but no detectable Aβ pathology [21]. The current cohort of cases provides an opportunity to evaluate the earliest deposition of AT8+ and TNT2+ tau pathology deposition in the DG-mossy fiber and CA3-Schaffer collateral pathways of the hippocampus. Cases were separated into PART (global plaque density = 0) or non-PART groups (global plaque density > 0). Both PART and non-PART groups contained 9 Braak I-II stage cases, while the PART group contained only 4 Braak stage III and the non-PART group contained 22 Braak stage III cases. Non-PART cases showed significantly more AT8+ neurites in the CA3 Str. Luc. (mossy fibers; Fig. 9b; *p* = 0.0005) and CA1 Str. Rad. (Schaffer collaterals; Fig. 9d; *p* = 0.0095), as well as AT8+ cells in the DG (Fig. 9a; *p* < 0.0001) and CA3 (Fig. 9c; *p* = 0.0011) neuron layers (Mann-Whitney U tests). Non-PART cases showed significantly more TNT2+ neurites in the CA3 Str. Luc. (mossy fibers; Fig. 9f; *p* = 0.0021) and CA1 Str. Rad. (Schaffer collaterals; Fig. 9h; *p* = 0.0001), but TNT2+ cells in the DG (Fig. 9e; *p* = 0.0965) and CA3 (Fig. 9g; *p* = 0.4557) neuron layers were not significantly different between PART and non-PART cases (Mann-Whitney U tests). Interestingly, DG neurons appear relatively spared in PART cases compared to non-PART cases as several lacked detectable somata pathology.

## Discussion

A long-held hypothesis in the field is that pathological tau deposition in disease begins in axons and progressively shifts retrogradely to the somatodendritic compartment [6, 14], but to our knowledge, no evidence from relatively discrete intrahippocampal neuronal pathways was available previously. The present study characterized the localization of mossy fiber and Schaffer collateral pathway tau pathology in a cohort of ND and MCI human cases. Our focus on relatively discrete neuronal pathways within in the hippocampus provides an opportunity to dissect the spatial changes that occur in the axonal and cell body compartments of neurons with some degree of specificity. Moreover, the use of Braak I-III cases with a range of local pathology load was instrumental in uncovering the first detectable deposition of tau inclusions in cell bodies the hippocampal pathways analyzed, which is reflected in the lack of local pathology and sparseness of pathology in several cases. Two early pathological markers in tauopathies, AT8 phosphorylation [9, 12, 32] and PAD exposure (TNT2) [18, 19], appeared in the axonal compartment of these pathways, even in cases where no cell body pathology was observed. Additionally, there was a strong correlation between local axonal pathological staining and the local number of stained cell bodies. Local AT8 and TNT2 positive neurite pathology also correlated well with overall indices of AD-related pathology such as Braak staging, global tangle density and NIA-Reagan level (particularly in the Schaffer collateral pathway). It is noteworthy that other hippocampal formation pathways such as the CA1 projections and EC-perforant pathway were not usable for the purposes of this study because both cell body and axonal pathology already existed in all cases. Taken together, these results support the hypothesis that tau pathology, at least in the studied pathways, is first observed in the axonal compartment and subsequently progresses into the somatodendritic compartment.

The purpose of this study was specifically to capture the earliest possible signs of pathological tau deposition within well-defined circuits to establish whether there is a difference in the temporal appearance of tau pathology in the axonal or cell body compartments of affected neurons. While the results indicate that PAD exposed (TNT2) and AT8 pathological changes occur early in disease progression, in post-mortem human tissue studies we cannot rule out the possibility that the tau deposition here is independent of a progressive condition that would have definitively converted to AD. Indeed, cohorts of MCI patients from previous studies clearly indicate that some patients do not ultimately convert to AD [62]. More recently, AD-like limbic tau pathology in the absence of Aβ pathology was termed primary age-related tauopathy (PART) [21], a condition typically associated with no to mild impairments. Here, we assessed the local accumulation of AT8 and TNT2 pathology in the DG-mossy fiber and CA3-Schaffer collateral pathways of the hippocampus. When cases were separated into potential PART (i.e. no Aβ patholoy) or non-PART (i.e. with Aβ pathology) groups the majority of measures found significantly more tau pathology in non-PART cases. While these findings could suggest Aβ exacerbates the deposition of these tau pathologies in these specific intrahippocampal pathways it is important to note that the non-PART group contained substantially more Braak stage III cases compared to the PART group. The only additional notable distinction between PART and non-PART groups was the lack of AT8+ DG neurons in PART cases suggesting DG neurons may be relatively spared from tau deposition in the early stages of PART progression.

Similar to synaptic loss [8, 23], the extent of total tau burden within the temporal lobe and hippocampus in neuroimaging and neuropathological studies shows a high degree of correlation with cognitive decline [30, 51, 70]. Interestingly, MMSE scores did not significantly correlate with any of the measures of local AT8+ and TNT2+ pathology in the DG-mossy fiber or CA3-Schaffer collateral pathways. It is likely that the extent of pathology in these discrete hippocampal strata represents the earliest possible stage of detection and thus below the threshold for an association with overt functional impairment, which is relatively mild in this cohort of cases. Case selection for this study specifically excluded those with dementia, limiting the range of MMSE scores available for correlation. The more extensive tau burden within temporal lobe and medial temporal lobe structures that contain a larger tau burden (e.g. the entorhinal cortex-perforant pathway) are likely better indices of cognitive decline in these early prodromal AD stages. This aligns with findings that other temporal lobe and medial temporal lobes are more severely affected when compared to CA3 and dentate neurons [27].

Synaptic loss and axon dysfunction begin in the prodromal stages of AD (i.e. MCI) and continue as AD progresses and these neuropathological events also occur in other tauopathies [8, 23, 66–68]. The high level of colocalization between AT8 and TNT2 with the axon-specific antibody SMI-312, but not the dendritic marker MAP2, further supports our conclusion that we evaluated predominantly axonal pathology. Additionally, several tau-positive neurites were not clearly colocalized with either SMI-312 or MAP2 making their origin difficult to determine. This could be the result of technical problems such as poor multi-labeling in the relatively small neurites containing densely packed tau inclusions. Alternatively, the loss of SMI-312 or MAP2 could be due to biological processes including degradation of cytoskeletal components such as neurofilaments that occurs during axonal degeneration [16, 17]. Though these issues preclude definitive identification of all tau-positive neurites in the tissue our data confirm that many of the neurites are axonal in origin (as indicated by the SMI-312 marker) within these specific hippocampal regions.

The presence of both TNT2 and AT8 positive tau pathologies in axons early in disease has important functional implications. The N-terminal PAD domain of tau (i.e. amino acids 2–18) was identified as a biologically active motif that when aberrantly exposed inhibits anterograde fast axonal transport in squid axoplasm [41, 48]. Additionally, the underlying molecular pathway of the axonal dysfunction was PAD-mediated activation of a protein phosphatase 1-glycogen synthase kinase-3β (PP1-GSK3β) signaling cascade [40, 42, 58, 59]. Our observation that AT8 pseudophosphorylation structurally exposes PAD aligns with prior structural studies using FRET assays [38], and we previously demonstrated that AT8 tau is toxic to axonal transport [41]. Thus, the appearance of PAD exposed tau and AT8 tau first in the axonal compartment of affected neurons before the observation of somatodendritic pathologies and the emergence of clinical symptoms further supports the hypothesis that the tau-mediated activation of the PP1-GSK3β signaling cascade may represent a relevant mechanism of neurodegeneration in tauopathies [42, 60]. Additionally, PAD exposure is a common occurrence across a range of tauopathies beyond AD, such as frontotemporal lobar degeneration [19]. Several pathological modifications of tau can contribute to PAD exposure, including phosphorylation [38, 73], oligomerization, and aggregation [20, 39]. Therefore, our observations that pathological modifications of tau previously shown to cause axonal dysfunction suggests that this may be one of the early tau-based mechanisms of degeneration in AD.

Our data now provide human tissue-based evidence supporting the hypothesis that tau deposition can occur in axons prior to the somata, but the nature of human tissue studies does not clarify whether the axonal tau pathologies are mobile and traverse retrogradely to the somata or are generated locally in each compartment. Originally, tau was thought to be an axon-specific protein [65], however, follow-up studies clearly found that tau is present throughout the neuron and only somewhat enriched in axons [10, 49]. Under disease conditions, redistribution of tau from the axon to the somatodendritic compartment is thought to be an important early event in pathogenesis. Additionally, phosphorylation of tau at residues known to alter tau’s conformation in disease are localized to the somatodendritic compartment, including AT8 [15, 38, 43]. The issue of whether tau pathologies traverse through neurons in any direction is likely going to be difficult to determine from human studies, however, a number of model systems suggest that pathological tau species are mobile within neurons. For example, a study investigating tau localization in neurons found that P301L mutant tau redistributed from the axonal compartment to the somatodendritic compartment in both transgenic mice expressing P301L tau and wild-type rat hippocampal primary neurons [37]. In addition, rat primary hippocampal neuron cultures exposed to Aβ oligomers showed an increased redistribution of tau to the somatodendritic compartment, and subsequent analyses found an increase in phosphorylation, including at the AT8 epitope, of this somatodendritic tau compared to untreated cultures [78], indicating the distribution of pathological tau can change in neurons. Although it is of interest to know whether these events occur in humans, independent of knowing the mechanisms of tau spread, the current work places forms of tau that are toxic to axonal function in the axons of neurons prior to the cell body in the early stages of tau deposition.

Notably, we observed local AT8+ and TNT2+ tau pathology independent of MOAB2+ Aβ pathology. The amyloid cascade hypothesis suggests that amyloid pathology occurs first in AD, and that tau pathology is a downstream consequence of Aβ pathology [35]. We observed evidence to the contrary; AT8 and TNT2 tau pathology occurred in the hippocampus in the absence of Aβ plaque pathology. Our data clearly indicate that overt Aβ pathology is not present despite the presence of pathological tau accumulation in the axon that was quite robust in some cases, thereby demonstrating a disconnection between Aβ plaque deposits and the emergence of tau inclusions. While it is possible that sections adjacent to those analyzed in this study could contain Aβ pathology in these regions, the lack of pathology in 55–84% of cases and the agreement of our results with the known spatiotemporal distribution of amyloid pathology (i.e. occurs first in neocortical regions) and tau pathology (i.e. occurs much later in neocortical areas) suggests the disconnect between tau and amyloid pathology is unlikely a result of our sampling [12, 14, 72]. The containment of the axonal compartment and cell bodies within discrete hippocampal strata provides a relatively clear demonstration that amyloid pathology in the terminal regions of tau-affected neurons does not necessarily occur in the pathways/regions assessed. Our findings align with previous findings by Braak and colleagues (1994) that very little to no Aβ pathology was present despite the presence of AT8 tau pathology within the transentorhinal/EC regions of Braak stage 0-III cases [12]. More recently, Lace et al. demonstrated that Aβ pathology was not correlated to AT8 tau pathology in the entorhinal cortex in a cohort of 93 cases ranging from nondemented to AD and Braak stages I-VI [47]. Additionally, we found that 29.5% of our cases displayed tau pathology without Aβ pathology and Lace et al. found this in 20% of their cases [47].

Amyloid plaques are thought to precipitate via a sequential process of going from monomeric proteins, to oligomeric species and then fibrillar forms [63]. The MOAB-2 antibody is a pan-Aβ specific antibody that reacts with monomeric, oligomeric and fibrillar forms of Aβ [77], suggesting that the tau pathologies observed in these pathways were not associated with pre-fibrillar or fibrillar forms of Aβ pathology. Again, we cannot completely rule out the possibility that some species of Aβ were not effectively detected or do not exist in other hippocampal regions or these specific pathways that contribute to tau accumulation without examining the entire region of interest. Additional studies assessing the time course of the spatiotemporal distribution of various Aβ and tau species may further clarify the relationship between these two hallmark AD pathologies. However, the data presented here directly challenge the proposition that aggregated Aβ triggers tau pathology in the Schaffer collateral and mossy fiber pathways within the hippocampus.

Overall, this study provides strong evidence that two early tau pathological markers, AT8 phosphorylation and PAD exposure, appear first in axons followed by deposition of cell body pathology. To our knowledge, this is the first study to systematically investigate two well-defined pathways within the hippocampus to differentiate between axon enriched strata and the corresponding cell bodies. Furthermore, visualizing these pathologically relevant modifications of tau in ND and MCI human tissue cases provides valuable insight into early tau pathology in the human condition before the onset of AD. Importantly, both AT8 and PAD exposed forms of tau are linked to mechanisms of toxicity in axonal functions such as axonal transport. Coupling these results with the lack of observable Aβ pathology suggests amyloid is not causing the early tau changes in these hippocampal pathways. This raises questions of whether the amyloid cascade is a viable hypothesis to explain the complex nature of AD etiology, but perhaps later in the disease process tau and Aβ pathologies work together to enhance ongoing cell dysfunction and degeneration once the pathologies overlap or interact. Nonetheless, our findings suggest that early axonal tau pathologies may trigger degenerative events in the hippocampal circuitry prior to overt cognitive decline, and more longitudinally focused future studies specifically on connecting the earliest forms of tau pathology in axons and clinical decline are needed.

## Additional files

Additional file 1: **Figure. S1.** Primary delete control experiment of antibodies used in IHC experiments. The same case was used for each staining and images were obtained in the same cortical gyrus. (a) AT8-labeled, (b) TNT2-labeled, and (c) MOAB2-labeled sections show positive immunoreactivity with each antibody. (d) Section stained with all components used in the IHC technique except the primary antibody resulted in no development of IHC signal, indicating the signals obtained in sections containing primary antibody are not due to non-specific reactivity or background signal from the tissue. Scale bar in (d) is 100 μm and applies to all panels. (TIF 2390 kb)

Additional file 2: **Figure S2.** Primary delete control experiment of antibodies used in AT8-TNT2 double label immunofluorescence experiments. The same case was used for each staining and all images were obtained in the same cortical gyrus. (a) Representative image of a section lacking the TNT2 primary antibody shows no cross reactivity with AT8 antibody labeling. (b) Representative image of a section lacking AT8 primary antibody shows no cross reactivity with TNT2 antibody labeling. These results confirm the specificity of AT8 and TNT2 co-localization in Fig. 5. Scale bars are 25 μm. (TIF 4800 kb)

Additional file 3: **Figure S3.** Primary delete control experiment of antibodies used in AT8/SMI-312/MAP2 and TNT2/SMI-312/MAP2 triple-label immunofluorescence experiments. The same case was used for each staining and all images were obtained in the hippocampus (CA1 region depicted). (a) Representative image of a section lacking the TNT2 primary antibody shows no cross reactivity with SMI-312 or MAP2 antibody labels. (b) Representative image of a section lacking AT8 primary antibody shows no cross reactivity with SMI-312 or MAP2 antibody labels. These results confirm the specificity of AT8 and TNT2 colocalizaiton with SMI-312 in Figs. 6 and 7. Scale bars are 25 μm. (TIF 5750 kb)

Additional file 4: **Figure S4.** AT8+ and TNT2+ neurite pathology in the DG-mossy fiber pathway does not change with clinical diagnosis or sex. (a-b). No significant differences in the AT8+ (a; *p* = 0.1325) or TNT2+ (b; *p* = 0.4115) neurites of the CA3 Str. Luc. layer when cases were compared across diagnosis group (ND, *N* = 31; MCI, *N* = 13). (c-d). No significant differences in the AT8+ (c; *p* = 0.3111) or TNT2+ (d; *p* = 0.8963) neurites of the CA3 Str. Luc. layer when cases were compared by sex (male, *N* = 27; female, *N* = 17). All comparisons made using Mann-Whitney U-test and the data are median with interquartile range. (TIF 5370 kb)

Additional file 5: **Figure S5.** AT8+ or TNT2+ neurite pathology in the CA3-Schaffer collateral pathway does not change with clinical diagnosis or sex. (a-b). No significant differences in the AT8+ (a; *p* = 0.854) or TNT2+ (b; 0.3054) neurites of the CA1 Str. Rad. layer when compared across diagnosis group (ND, *N* = 31; MCI, *N* = 13). (c-d). No significant differences in the AT8+ (c; *p* = 0.5337) or TNT2+ (d; *p* = 0.2268) neurites of the CA1 Str. Rad. layer when compared across sex (Male, *N* = 27; Female, *N* = 17). All comparisons made using Mann-Whitney U-test and the data are median with interquartile range. (TIF 4510 kb)

Additional file 6: **Figure S6.** AT8+ and TNT2+ tau neurite pathology colocalizes with the axonal marker SMI-312 in the Schaffer collateral and mossy fiber pathways, respectively. (a-b) Representative images of TNT2 (green), SMI-312 (red) and MAP2 (cyan) triple labeling immunofluorescence staining in the mossy fiber pathway of the hippocampus (merged image in a includes DAPI nuclear counter stain). (a) A low magnification image shows axonal (SMI-312), dendritic (MAP2) and tau+ neurites (TNT2) in the CA3 Str. Luc. region of the hippocampus in a Braak stage III case. (b) Cross-sectional analysis of z-stack images (60x magnification 0.5 μm step size) demonstrate that TNT2+ neurites colocalize with SMI-312 (arrows). (c-d) Representative images of AT8 (green), SMI-312 (red) and MAP2 (cyan) triple labeling immunofluorescence staining in the Schaffer collaterals of the hippocampus (merged image in a includes DAPI nuclear counter stain). (c) A low magnification image shows axonal (SMI-312), dendritic (MAP2) and tau+ neurites (AT8) in the CA1 Str. Rad. region of the hippocampus in a Braak stage III case. (d) Cross-sectional analysis of z-stack images (60x magnification 0.5 μm step size) demonstrate that AT8+ neurites colocalize with SMI-312 (arrows). Notably, little to no colocalization was observed between AT8+ or TNT2+ neurites and MAP2. Scale bars are 25 μm for a and c, or 5 μm for b and d. See also Figs. 6 and 7. (TIF 1660 kb)
