# Supplementary figures and images for: Pathogenic tau modifications occur in axons before the somatodendritic compartment in mossy fiber and Schaffer collateral pathways
Source: Acta Neuropathol Commun. 2019 Feb 28;7:29. doi: 10.1186/s40478-019-0675-9 (PMC6394076; doi:10.1186/s40478-019-0675-9)

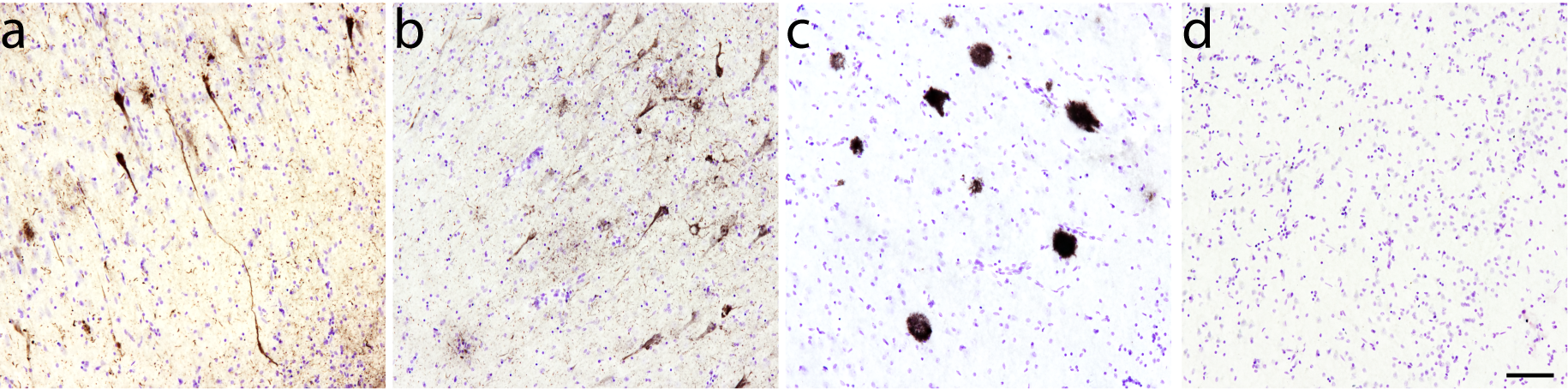

Supplement: Supplementary file 1 — Figure. S1. Primary delete control experiment of antibodies used in IHC experiments. The same case was used for each staining and images were obtained in the same cortical gyrus. (a) AT8-labeled, (b) TNT2-labeled, and (c) MOAB2-labeled sections show positive immunoreactivity with each antibody. (d) Section stained with all components used in the IHC technique except the primary antibody resulted in no development of IHC signal, indicating the signals obtained in sections containing primary antibody are not due to non-specific reactivity or background signal from the tissue. Scale bar in (d) is 100 μm and applies to all panels. (TIF 2390 kb) [file 40478_2019_675_MOESM1_ESM.tif]

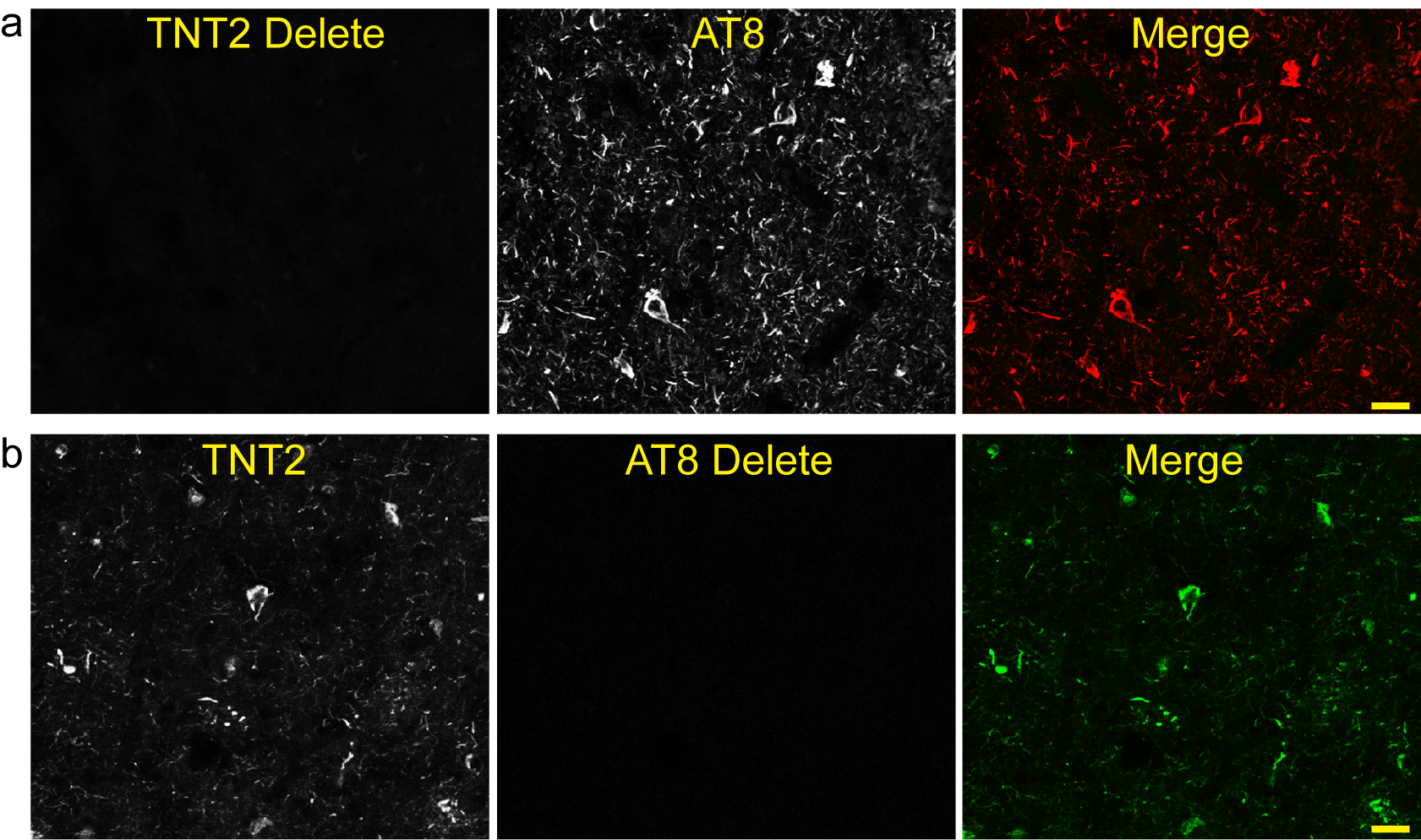

Supplement: Supplementary file 2 — Figure S2. Primary delete control experiment of antibodies used in AT8-TNT2 double label immunofluorescence experiments. The same case was used for each staining and all images were obtained in the same cortical gyrus. (a) Representative image of a section lacking the TNT2 primary antibody shows no cross reactivity with AT8 antibody labeling. (b) Representative image of a section lacking AT8 primary antibody shows no cross reactivity with TNT2 antibody labeling. These results confirm the specificity of AT8 and TNT2 co-localization in Fig. 5. Scale bars are 25 μm. (TIF 4800 kb) [file 40478_2019_675_MOESM2_ESM.tif]

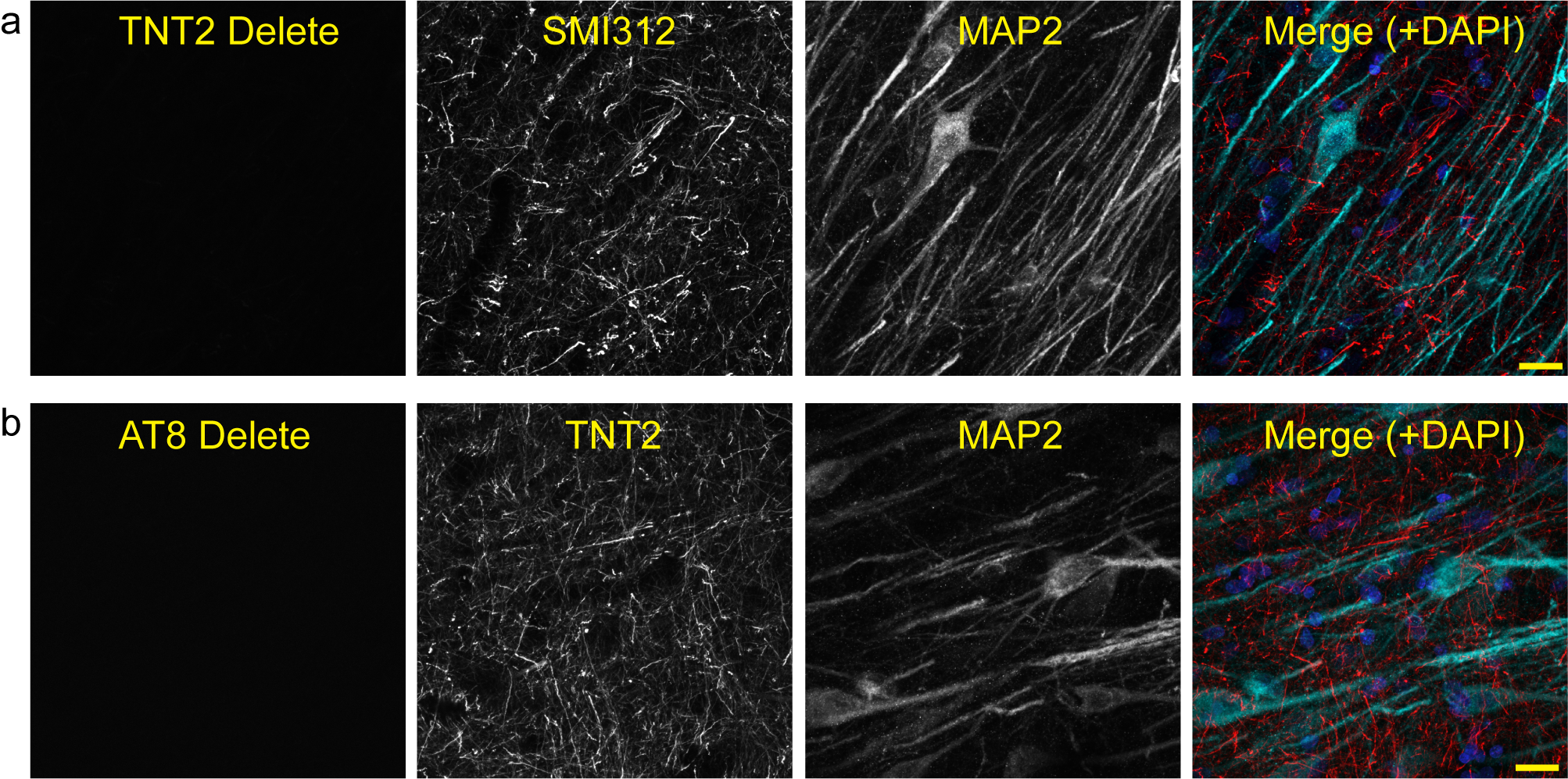

Supplement: Supplementary file 3 — Figure S3. Primary delete control experiment of antibodies used in AT8/SMI-312/MAP2 and TNT2/SMI-312/MAP2 triple-label immunofluorescence experiments. The same case was used for each staining and all images were obtained in the hippocampus (CA1 region depicted). (a) Representative image of a section lacking the TNT2 primary antibody shows no cross reactivity with SMI-312 or MAP2 antibody labels. (b) Representative image of a section lacking AT8 primary antibody shows no cross reactivity with SMI-312 or MAP2 antibody labels. These results confirm the specificity of AT8 and TNT2 colocalizaiton with SMI-312 in Figs. 6 and 7. Scale bars are 25 μm. (TIF 5750 kb) [file 40478_2019_675_MOESM3_ESM.tif]

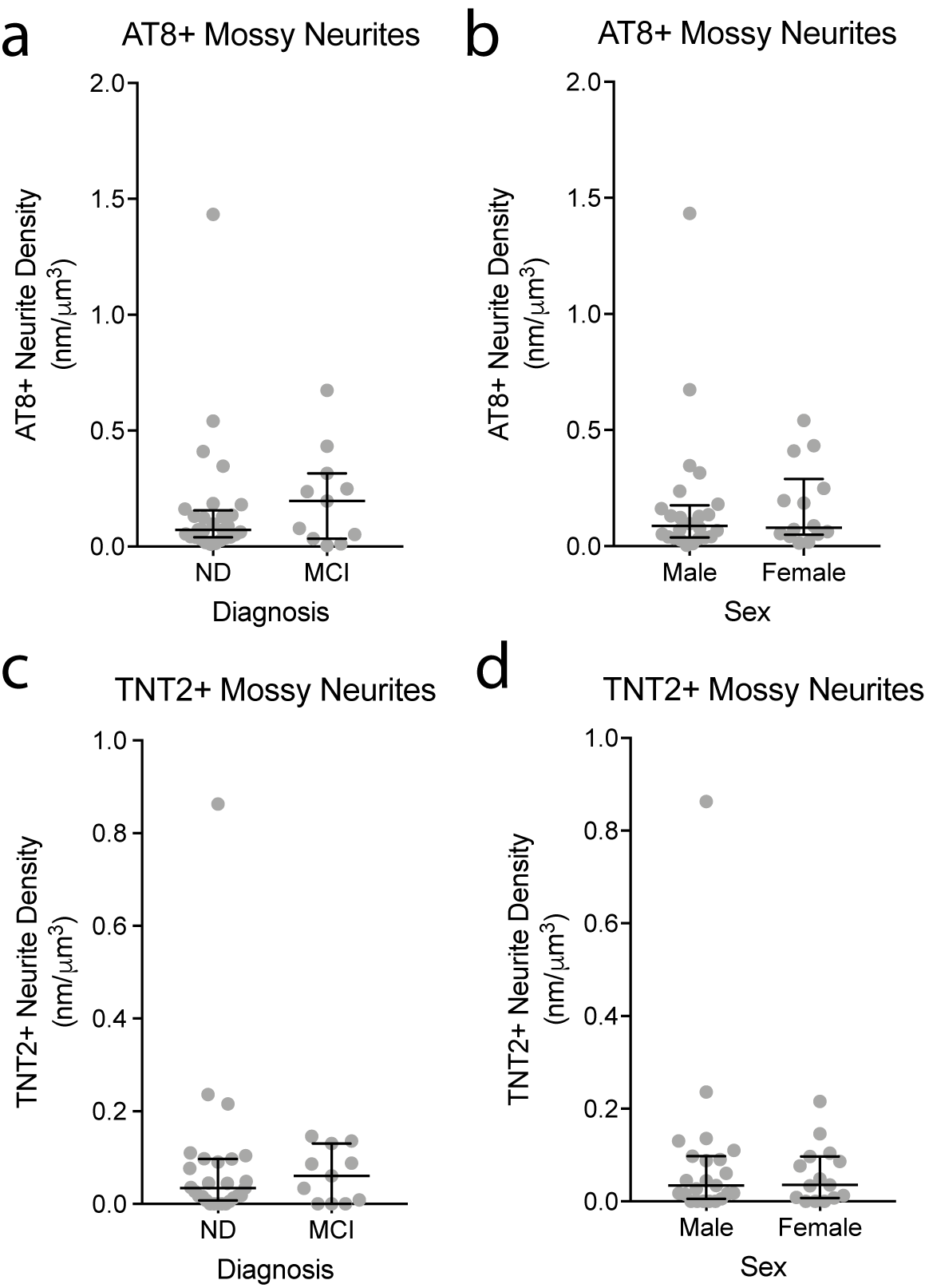

Supplement: Supplementary file 4 — Figure S4. AT8+ and TNT2+ neurite pathology in the DG-mossy fiber pathway does not change with clinical diagnosis or sex. (a-b). No significant differences in the AT8+ (a; p = 0.1325) or TNT2+ (b; p = 0.4115) neurites of the CA3 Str. Luc. layer when cases were compared across diagnosis group (ND, N = 31; MCI, N = 13). (c-d). No significant differences in the AT8+ (c; p = 0.3111) or TNT2+ (d; p = 0.8963) neurites of the CA3 Str. Luc. layer when cases were compared by sex (male, N = 27; female, N = 17). All comparisons made using Mann-Whitney U-test and the data are median with interquartile range. (TIF 5370 kb) [file 40478_2019_675_MOESM4_ESM.tif]

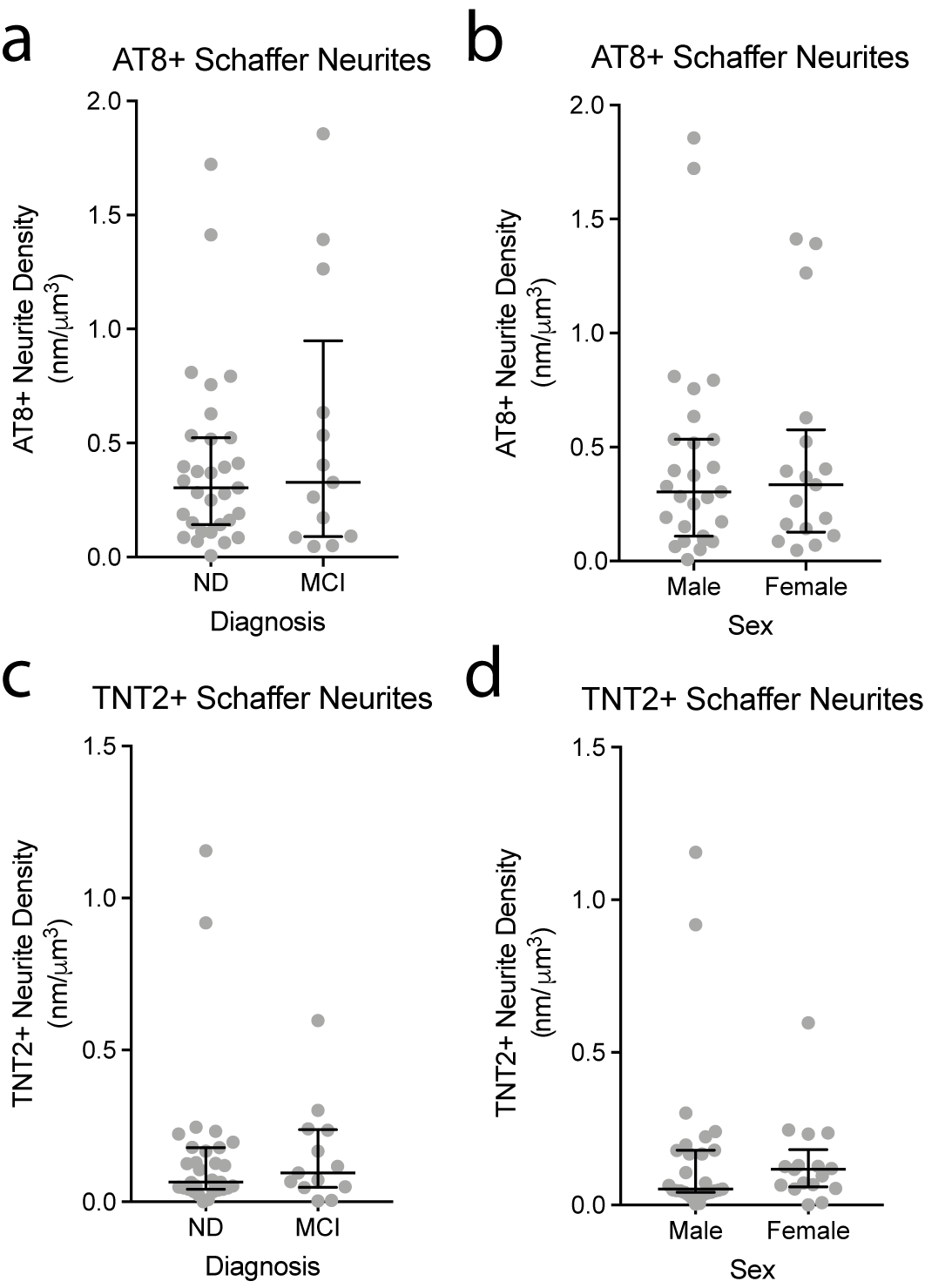

Supplement: Supplementary file 5 — Figure S5. AT8+ or TNT2+ neurite pathology in the CA3-Schaffer collateral pathway does not change with clinical diagnosis or sex. (a-b). No significant differences in the AT8+ (a; p = 0.854) or TNT2+ (b; 0.3054) neurites of the CA1 Str. Rad. layer when compared across diagnosis group (ND, N = 31; MCI, N = 13). (c-d). No significant differences in the AT8+ (c; p = 0.5337) or TNT2+ (d; p = 0.2268) neurites of the CA1 Str. Rad. layer when compared across sex (Male, N = 27; Female, N = 17). All comparisons made using Mann-Whitney U-test and the data are median with interquartile range. (TIF 4510 kb) [file 40478_2019_675_MOESM5_ESM.tif]

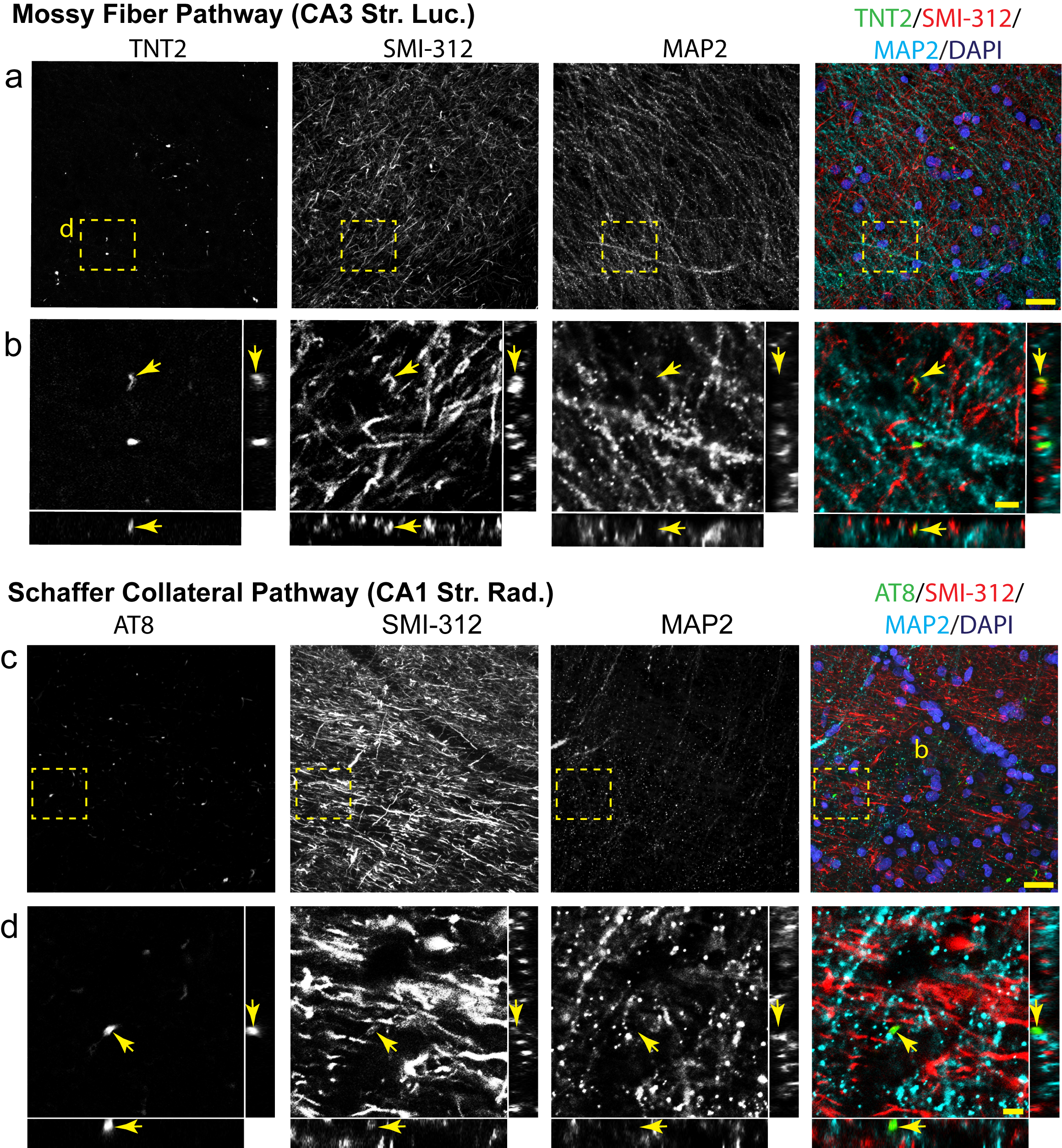

Supplement: Supplementary file 6 — Figure S6. AT8+ and TNT2+ tau neurite pathology colocalizes with the axonal marker SMI-312 in the Schaffer collateral and mossy fiber pathways, respectively. (a-b) Representative images of TNT2 (green), SMI-312 (red) and MAP2 (cyan) triple labeling immunofluorescence staining in the mossy fiber pathway of the hippocampus (merged image in a includes DAPI nuclear counter stain). (a) A low magnification image shows axonal (SMI-312), dendritic (MAP2) and tau+ neurites (TNT2) in the CA3 Str. Luc. region of the hippocampus in a Braak stage III case. (b) Cross-sectional analysis of z-stack images (60x magnification 0.5 μm step size) demonstrate that TNT2+ neurites colocalize with SMI-312 (arrows). (c-d) Representative images of AT8 (green), SMI-312 (red) and MAP2 (cyan) triple labeling immunofluorescence staining in the Schaffer collaterals of the hippocampus (merged image in a includes DAPI nuclear counter stain). (c) A low magnification image shows axonal (SMI-312), dendritic (MAP2) and tau+ neurites (AT8) in the CA1 Str. Rad. region of the hippocampus in a Braak stage III case. (d) Cross-sectional analysis of z-stack images (60x magnification 0.5 μm step size) demonstrate that AT8+ neurites colocalize with SMI-312 (arrows). Notably, little to no colocalization was observed between AT8+ or TNT2+ neurites and MAP2. Scale bars are 25 μm for a and c, or 5 μm for b and d. See also Figs. 6 and 7. (TIF 1660 kb) [file 40478_2019_675_MOESM6_ESM.tif]
